# Mind-Body Therapies for Depression and Anxiety Symptoms in People with Cancer: A Systematic Review with Network Meta-Analysis

**DOI:** 10.1007/s11912-026-01790-7

**Published:** 2026-05-18

**Authors:** Yoann Birling, Deep Jyoti Bhuyan, Fan Feng, Mingxian Jia, Jing Liu, Han Zhang, Matthew Rahimi, Wing Yu Yu, Linda E. Carlson, Hazel Betul Boge, Nibras Jasim, Kayla Jaye, Indeewarie Hemamali Dissanayake, Sarah Nevitt, Judith Lacey, Rogier Hoenders, Changrong Tang, Tiffany Tram, Paul P. Fahey

**Affiliations:** 1 NICM Health Research Institute, Western Sydney University, Locked Bag 1797, Penrith, NSW 2751 Australia; 2 Benson Henry Institute for Mind Body Medicine, Massachusetts General Hospital, Boston, MA USA; 3 Beijing University of Chinese Medicine, Beijing, China; 4 School of Health Sciences, Western Sydney University, Penrith, NSW Australia; 5 Woolcock Institute of Medical Research, Macquarie Park, Sydney, NSW Australia; 6 Western Sydney University, Penrith, NSW Australia; 7 University of Calgary, Calgary, AB Canada; 8 School of Psychology, Western Sydney University, Penrith, NSW Australia; 9 Department of Health Data Science, University of Liverpool, Liverpool, UK; 10 Centre for Reviews and Dissemination, University of York, Heslington, York UK; 11 Chris O’Brien Lifehouse Supportive Care and integrative oncology, Camperdown, NSW Australia; 12 Center for Integrative Psychiatry, Faculty of Religion, Culture and Society, University of Groningen, Lentis, Groningen, Netherlands; 13 Daxing Office of Beijing Institute of Health Supervision, Beijing, China

**Keywords:** Cancer, Oncology, Depression, Anxiety, Mind-body therapies, Mindfulness, Yoga, Relaxation

## Abstract

**Intro:**

Mind-body therapies (MBTs) are promising forms of treatment for depression and anxiety symptoms in people with cancer, however, their effectiveness has not been compared. The objective of this study is to rank the effectiveness of MBTs for depression and anxiety symptoms in people with cancer.

**Methods:**

EMBase, PubMed, Cinahl, PsychINFO, IndMED, CSI-NISCAIR, CNKI, Clinicaltrial.gov, ChiCTR, and CTRI were searched until February 2025 for randomised controlled studies in which MBTs were tested in a cancer population. Network meta-analyses were used on the selected studies to rank the reduction in depression and anxiety symptoms compared with usual care.

**Results:**

A total of 182 studies which involved 16,835 participants were included. The network meta-analysis showed a statistically significant large effect compared with usual care for biofeedback (standardised mean difference, SMD = -1.18, *p* = 0.038), spiritual interventions (SMD = -0.98, *p* = 0.047), and mindfulness-based interventions (MBIs; SMD = -0.87, *p* < 0.001) for depression symptoms and yoga (SMD = -1.13, *p* < 0.001), MBIs (SMD = -1.02, *p* < 0.001), physical relaxation (SMD = -0.93, *p* < 0.001), qigong/Tai Chi (QTC; SMD = -0.90, *p* = 0.004) and music therapy (SMD = -0.90, *p* < 0.001) for anxiety symptoms.

**Conclusions:**

The MBTs that should be prioritised are biofeedback, spiritual interventions, and MBIs for depression symptoms and yoga, MBIs, physical relaxation, QTC, and music therapy for anxiety symptoms in people with cancer.

**Supplementary Information:**

The online version contains supplementary material available at 10.1007/s11912-026-01790-7.

## Introduction

Depression and anxiety symptoms are highly prevalent in people with cancer, affecting approximately 20% to 30% of individuals in this population [1–4]. Psychological health strongly predicts quality of life in people with cancer [5] and and psychological symptoms impacts patient adherence to antineoplastic treatment, leading to poorer treatment outcomes [6–8]. Moreover, depression and stress are associated with inflammation and a weaker immune system, which may affect the prognosis of cancer [9–13]. For the above reasons, depression and anxiety symptoms are associated with increased cancer recurrence and mortality in people with cancer [14–16]. The effective management of depression and anxiety symptoms could improve both adherence to anticancer treatment and mortality rates [17–19].

Depression and anxiety symptoms in people with cancer are mostly treated with pharmacological drugs, such as antidepressants and anxiolytics [20, 21], which may be associated with adverse reactions, such as morning sedation, anterograde amnesia, falls, undesired sleep behaviour, gastrointestinal symptoms, weight gain, sexual dysfunction, and anxiety [22–24]. Moreover, long-term use of psychotropic medications may increase the risk of dementia, diabetes, cancer and overall mortality rates [25–29]. These adverse reactions encourage clinicians and researchers to look for alternatives such as mind-body therapies (MBTs) for the management of depression and anxiety symptoms in cancer patients.

MBTs are defined as “a group of healing techniques, either taught by a trained practitioner or self-guided, that target the mind (e.g., thoughts) and body (e.g., sensations) to enhance mental and physical health and well-being” [30]. Traditional MBTs such as yoga, meditation, Qi gong and Tai Chi, which are rooted in Hinduist, Buddhist, and Taoist traditions, originally share an element of spirituality with spiritual interventions such as prayer and religious chanting [31]. In the context of Western healthcare, these interventions are usually offered with little or none of these spiritual aspects [32]. Similarly, mindfulness, often defined as non-judgmental, present-focused awareness of one’s thoughts, feelings, and bodily sensations, stems from Buddhist teaching and philosophy, but has been secularised and popularised in the West through the structured Mindfulness-Based Stress Reduction (MBSR) program originated by Jon Kabat-Zinn [33]. Adaptations of MBSR, such as Mindfulness-Based Cognitive Therapy (MBCT) [34], collectively are known as mindfulness-based interventions (MBIs) [30].

Some MBTs, such as relaxation training with or without imagery, have more emphasis on inducing the physiological relaxation response, which is an autonomic nervous system response opposed to the fight-or-flight stress reaction [35]. Creative therapies such as art, music and dance therapy help people cope with and process difficult emotions through nonverbal creative expression, which often enhances emotional awareness and acceptance and promotes adaptive emotion regulation [36]. Social support in many MBTs, typically offered in professionally led group formats, can counteract loneliness and feelings of isolation, which are common mediators of psychological symptoms experienced by people living with cancer [37]. Enhancing physical activity and interoceptive body awareness are other potentially effective components of MBTs [38, 39].

Systematic reviews have concluded that MBTs can reduce depression and anxiety symptoms [40, 41]. The mechanisms of these effects are likely multicomponent and may vary depending on the type of MBT. For example, activating the relaxation response, which is a common component of MBTs, involves decreasing metabolism, heart rate, and respiratory rate, and counteracts the effects of the stress reaction on health [35]. Neurological studies show that MBTs can affect brain structure and activity, including enhancement of neuroplasticity and an increase in low-frequency brain wave [39, 42]. MBTs decrease the expression of inflammation-related genes, leading to a reduction in systemic inflammation, which is known to be a mediator of depression and anxiety symptoms [43]. Musculoskeletal exertion and increased cardiovascular output can regulate the hypothalamus-pituitary-adrenal axis activity and sympathetic/parasympathetic balance [39]. Cognitively, MBIs, for example, have been shown to decrease rumination, worry and experiential avoidance, which are maladaptive emotion regulation strategies tied to increased rates of depression and anxiety [44].

Systematic reviews have shown that MBTs can improve the moods of people with cancer [45–50]. Both the European Society of Medical Oncology and the American Society of Clinical Oncology (ASCO) recommend MBIs for depression and anxiety symptoms in people with cancer [51, 52]. This shows that these therapies are progressively gaining acceptance. The Society for Integrative Oncology (SCO) and the ASCO jointly produced guidelines on the use of integrative approaches to managing anxiety and depression symptoms in people with cancer [53].

A myriad of MBTs are now available to people with cancer, ranging from relaxation and imagery, to hypnosis, yoga, meditation, Tai Chi and Qigong, and creative therapies [30]. This variety results in the dilemma of selecting the best intervention for the patient. For example, an oncologist may have the option to refer their patient to a MBI program or a yoga program and might not know which one is the best for them. The aforementioned systematic reviews did not compare the relative effectiveness of MBTs and the guidelines from the SCO and ASCO did not base their recommendations on the relative effectiveness of the MBT that were tested in clinical trials. This gap in the literature prevents clinicians from referring their patients to receive the best available intervention for their depression and anxiety symptoms.

The present review aims to fill the gap in the literature by providing a network meta-analysis (NMA) of the randomised-controlled trials that assessed the effectiveness of MBTs for depression and anxiety symptoms in people with cancer.

## Materials & Methods

This study was conducted according to the Cochrane guidelines on NMA systematic reviews [54]. The protocol was registered in International Prospective Register of Systematic Reviews (CRD42021240595). The Human Research Ethics Committee of Western Sydney University, Australia exempted the present review from ethical review. We originally aimed at conducting an individual participant data (IPD) review, however, due to the lack of data availability we decided to conduct the main analysis with aggregated data and present the IPD analysis in a secondary analysis. We also aimed originally at including insomnia symptoms, however, the NMA on insomnia symptoms was not completed due to constraints of time and resources. More details about the methods are available in the published protocol [55].

### Study Selection

#### Study Design

Studies in which participants were randomly allocated were included. Both parallel and cross-over studies were included. For cross-over studies, only the first period of the study was included.

#### Participants

We included adults diagnosed with cancer—regardless of the type of cancer, cancer stage, type of treatment received, or stage of treatment. However, studies conducted specifically around a medical procedure (e.g., biopsy and surgery) were excluded.

#### Interventions

MBI including MBSR as well as Mindfulness-Based Cancer Recovery (MBCR) and Mindfulness-Based Cognitive Therapy (MBCT), which are both developed from MBSR, yoga-based interventions, Tai Chi, qigong, visualization and imagery, hypnosis and self-suggestion, art therapy, music therapy, dance therapy, biofeedback, spiritual interventions (such as prayer and religious rituals), touch therapy, and relaxation training—including compound interventions of the above—were included in this review. Psychotherapy, which focuses heavily on the mind rather than the body, exercise therapy, which focuses mostly on the body, and manual therapies such as acupuncture and massage, which rely on techniques applied to the patient physically rather than engaging the patient mentally in the intervention, were not included. This is standard classification for these integrative therapies. Compound interventions, including MBT and counselling, education, and/or diet, were included only in cases where the MBT interventions were major.

#### Controls

Inactive and active controls, such as wait-list, no-treatment, usual care, exercise therapy, psychoeducation, sham MBT, or another MBT, were considered as appropriate control interventions.

#### Outcomes

Studies in which the depression and anxiety symptoms were assessed with a validated questionnaire were included, even where depression and anxiety symptoms were not the primary outcomes of the original study. The list of questionnaires accepted in this review is presented in Online Resource 1. The secondary outcome was the acceptability of the intervention, represented by the completion rate (i.e., the ratio of number of participants completing post-intervention assessments to the number of randomized participants).

### Search Strategy

EMBase, PubMed, Cinahl, Cochrane Library, PsychINFO, IndMED, CSIR-NISCAIR, CNKI, Clinicaltrial.gov, ChiCTR, and CTRI were searched from database inception until October 2020 (initial search) and until August 2022, August 2023, and February 2025 (follow-up searches). The search terms were constructed around four themes (i.e., “mental health”, “cancer,” “mind-body therapies,” and “randomized controlled trial”) using both medical heading terms and text words. The theme “mental health” covers insomnia, depression, and anxiety. No language restrictions were applied to the search. The full search strategy is reported in Online Resource 2. A total of 111 previous systematic reviews were hand-searched for additional studies and a forward search (i.e., the search of publications that cited the included articles) was conducted using PubMed.

### Screening

The studies were screened by five teams of two reviewers with each member of the pair working independently. The first step was title/abstract screening. Then the full text of each reference which may have been relevant following the title/abstract screening was downloaded. Finally, the full text was screened. The results of the screening were compared between the two reviewers of each team. Disagreements were resolved through discussion and then through the decision of a third reviewer where consensus was not reached.

### Data Extraction

A standardized data collection spreadsheet was used for data extraction. All data were reviewed by a second reviewer. Disagreements between the team member who extracted the data and the reviewer were all resolved through discussion or by consulting a third reviewer. The data collection spreadsheet included the following items:

- List of authors.
- Year of publication.
- Country in which the trial was conducted.
- Treatment arms with number of participants randomized and number of participants who completed the intervention period.
- Selection according to depression diagnosis and diagnostic tool, if applicable.
- Participant characteristics (sex, age, type and stage of cancer).
- Intervention details (intervention name, treatment frequency and duration, session duration, mode of delivery, presence of home practice) for experimental group.
- Outcome measures.
- Outcome at baseline, during treatment, and at post-treatment.

### Risk of Bias Assessment

Risk of bias was assessed by five teams of two reviewers working independently with version two of the Cochrane Collaboration’s Risk of Bias assessment tool [56]. When one study included both active and inactive control interventions, the risk of bias was assessed separately for each control intervention. Disagreements were resolved through discussion and consulting a third team member reviewer. We assessed potential publication bias with a funnel plot and the Egger’s test [57] for the primary meta-analyses.

### Heterogeneity and Consistency Assessment

The heterogeneity of the included studies was presented with the I^2^ statistic, with values of 50% or more considered to be indicators of a substantial level of heterogeneity [54]. A high level of heterogeneity was expected due to the wide range of MBTs and the diversity of population included in this meta-analysis. The reasons for the heterogeneity were explored through subgroup analyses and meta-regressions, yet a detailed exploration of the reasons for the heterogeneity and its impact on treatment outcomes is beyond the scope of this meta-analysis due to lack of access to IPD. Where high levels of heterogeneity remain unexplained, the reader is advised that some caution is warranted in interpreting results.

In order to comply with the transitivity assumption, only trials in which the interventions could be “jointly randomizable” [58] were included in the NMA. Inconsistency between direct and indirect sources of evidence was statistically assessed through the calculation of the differences between direct and indirect estimates in all closed loops in the network.

### Network Meta-Analyses

Interventions were grouped by name/title of the intervention (e.g., “yoga”, “qigong”) provided by the original authors of the study rather than treatment components (e.g., breathing techniques, mindfulness). For example, two interventions respectively labeled “yoga” and “qigong” by the study authors but involving the same components (e.g., breathing techniques and posture) were grouped under two different categories. Details on the interventions grouped together are shown in Online Resource 3. We conducted a subgroup analysis of the network analysis, in which interventions falling under the same umbrella, yet having some distinct features (e.g., imagery with or without virtual reality) were analysed separately.

In order to rank the effectiveness and acceptability of each MBT, an NMA was conducted. This NMA included direct and indirect comparisons between different MBTs. The NMA was adjusted for the presence of multi-arm trials. A network plot was used to present the geometry of the treatment network. Results were reported with SMD for depression and anxiety symptoms and RR for acceptability, both with 95% CIs. To rank the treatments for each outcome, the surface under the cumulative ranking (SUCRA) probabilities were used. Effect sizes of SMD > 0.8 were considered high [59].

All tests were 2-tailed, and a *p*-value of less than 0.05 was considered statistically significant. Statistical analyses were performed using RevMan v5 (for pairwise meta-analyses) and R v4.2.3 statistical software (for subgroup analyses, meta-regressions and network meta-analyses) using the packages metafor() and robumeta() during the meta-analyses and netmeta() during the network meta-analysis.

### Subgroup Analyses, Meta-Regressions and Sensitivity Analyses

Based on the primary meta-analysis, we conducted subgroup analyses and meta-regressions to assess the influence of the following factors on the reduction of depression symptoms:

- Cancer site.
- Cancer stage, separating early stages (stages 0, I and II) and late stages (stages III, and IV).
- Sex.
- Sample reaching or not the threshold of clinical depression and anxiety at baseline.
- Treatment duration.
- Treatment frequency.
- Total exposure to intervention.
- Intervention format: Group or individual.
- Intervention format: Supervised or unsupervised.
- Intervention format: Face-to-face or remote.
- Intervention format: Presence or absence of home practice.

Due to the lack of studies with a low risk of bias and the absence of studies in which depression and anxiety were diagnosed according to a recognized diagnostic standard, we could not conduct a sensitivity analysis with these studies as planned in the original protocol.

### IPD Analysis

A meta-analysis was conducted to test the impact of interventions on depression symptoms in the studies where IPD was available. The meta-analysis was fit using a random slopes mixed model. Preliminary diagnostic analyses using linear regression identified the potential for outliers and a skewed distribution of residuals. To address these potential problems we fitted robust models using the rlmer() command of the robustlmm package in R.

## Results

### Studies Characteristics

**Fig. 1:**
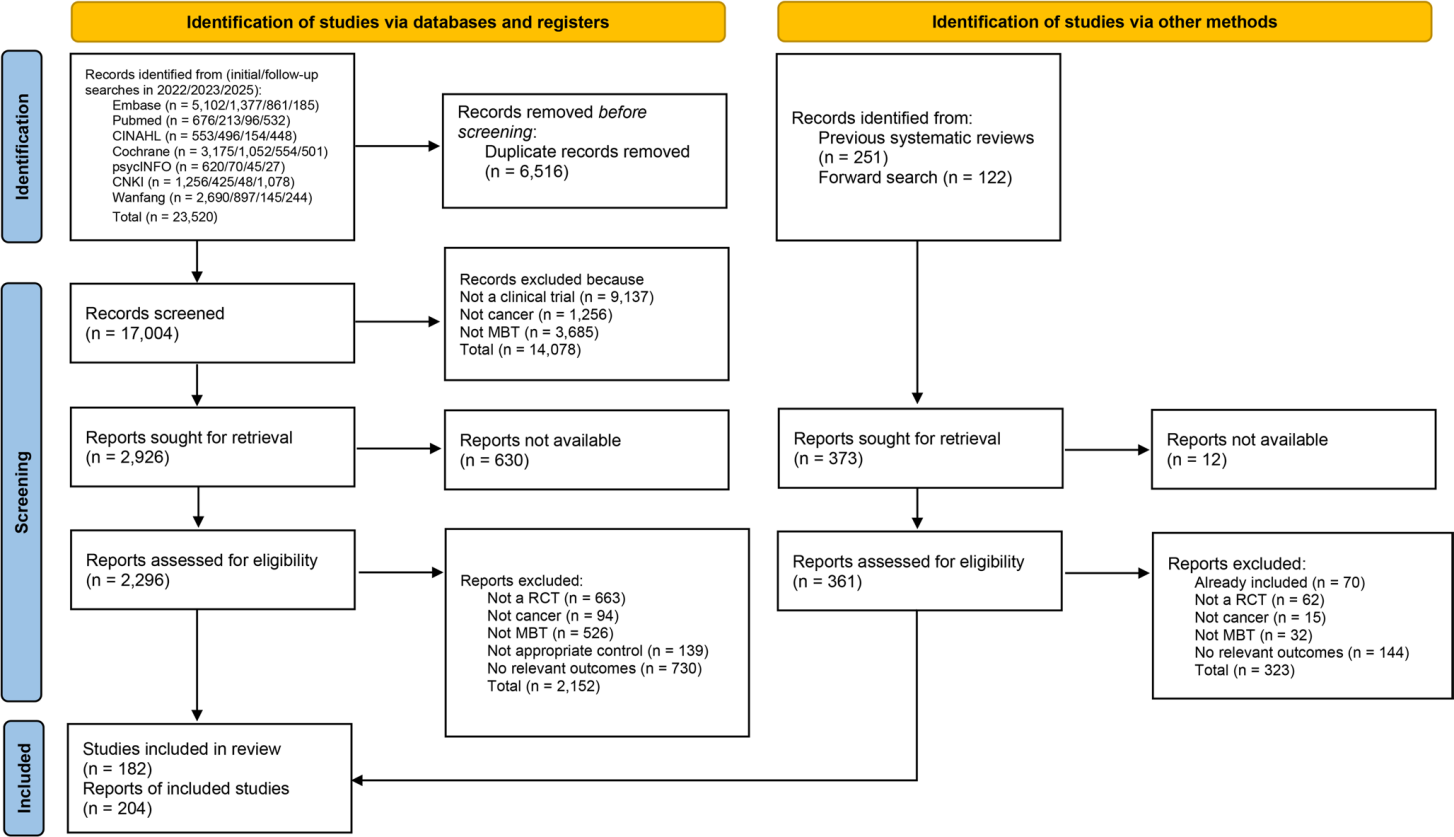
PRISMA flow diagram of the screening process

We included a total of 182 studies which involved 12,789 female participants, 3,380 male participants, and 666 participants of unknown sex. The PRISMA flow diagram of the screening process is reported in Fig. 1. The mean age of the participants was 53.0 years. The cancer type and cancer stage of the population included are reported in Table 1. The studies included 2 four-arm trials, 25 three-arm trials and 155 two-arm trials. The most tested MBTs were MBI (58 groups), music therapy (36 groups), and yoga (26 groups). The most common control interventions were usual care (94 groups) and waitlist (48 groups). A list of study characteristics for each study is available in Online Resource 4 and the dataset of this review is available in figshare at 10.6084/m9.figshare.28823228.

### Primary Meta-Analyses

A total of 113 studies in which MBT was compared with an inactive control provided depression symptoms at end-of-intervention. Overall, we found a statistically significant difference of SMD = −0.58 (95% CI −0.74 to −0.41, *p* < 0.001) in depression symptoms between mind-body therapies and the inactive control interventions. The test of heterogeneity showed significant heterogeneity, with a tau^2^ of 0.58 and a I^2^ value of 90.5%. Egger’s test (*p* = 0.351) and the funnel plot did not show evidence of publication bias.

A total of 106 studies comparing MBT with an inactive control provided anxiety symptoms at baseline and end-of-intervention. Overall, we found a statistically significant difference of SMD = −0.74 (95% CI −0.92 to −0.57, *p* < 0.001) in anxiety symptoms between mind-body therapies and the inactive control interventions. The test of heterogeneity showed significant heterogeneity, with a tau^2^ of 0.625 and a I^2^ value of 90.9%. Egger’s test (*p* = 0.025), but not the funnel plot, showed evidence of publication bias. The results of the assessment of risk of bias are presented in Online Resource 5.

A total of 146 studies in which MBT was compared with an inactive control and in which depression and anxiety were examined provided both number randomised and number completing in each treatment group. The estimated relative risk of completion in the MBT group was 0.989 times as large as the risk of completion in the control group (95% CI = 0.983 to 0.995, *p* = 0.001), which is statistically but not clinically significant.

### Subgroup Analyses and Meta Regressions

We examined the impact of population and intervention characteristics on treatment effectiveness. The results are presented in Table 1. The subgroup analyses and meta-regressions show that the effect of MBTs compared to control interventions is higher on patients with lung, blood and other cancers, mixed sex, and clinical depression/anxiety compared to patients with breast cancer, female sex, and no clinical depression/anxiety, respectively. The difference between patients with clinical depression/anxiety and patients with no clinical depression/anxiety was statistically significant. Interventions with a low session frequency (< 1/w) and remote interventions were less effective than other interventions, whereas interventions with very high total exposure (> 24 h) were more effective. Interventions using an individual format and the ones using supervision were slightly more effective (SMD = 0.09 to 0.24) than interventions using a group format and no supervision, respectively. Interventions with home practice were more effective for depression symptoms but less effective for anxiety symptoms than interventions without home practice.

**Table 1:** Subgroup analysis and meta-regression of MBT compared to control interventions on depression symptoms according to population and intervention characteristics. Asterisks refer to the statistical significance level, with * representing *p* < 0.05, ** representing *p* < 0.01, and *** representing *p* < 0.001

Characteristics	Depression symptoms				Anxiety symptoms	
	Groups	Sub-group SMD (95% CI)	Meta-regression SMD (95% CI)	Groups	Sub-group SMD (95% CI)	Meta-regression SMD (95% CI)
Cancer site						
Breast	55	−0.45 (−0.69, −0.21)***	Reference	43	−0.68 (−0.94, −0.42)***	Reference
Blood	5	−0.67 (−1.61, 0.28)	−0.22 (−1.16, 0.72)	8	−1.07 (−1.90, −0.25)*	−0.39 (−1.22, 0.44)
Lung	5	−1.56 (−2.84, −0.27)*	−1.11 (−2.35, 0.13)	6	−1.30 (−2.18, −0.41)*	−0.61 (−1.49, 0.26)
Other	18	−0.79 (−1.27, −0.31)**	−0.35 (−0.87, 0.18)	15	−0.85 (−0.94, −0.15)*	−0.17 (−0.90, 0.56)
Various	32	−0.53 (−0.84, −0.21)**	−0.08 (−0.47, 0.31) 8	41	−0.62 (−0.94, −0.31)***	0.06 (−0.34, 0.46)
Cancer stage						
Mostly stage 0–2	39	−0.50 (−0.78, −0.22)***	Reference	33	−0.68 (−1.00, −0.35)***	Reference
Mostly stage 3–4	19	−0.59 (−1.00, −0.17)**	−0.08 (−0.57, 0.40)	19	−0.79 (−1.27, −0.32)**	−0.12 (−0.67, 0.44)
Unknown	62	−0.62 (−0.87, −0.37)***	−0.11 (−0.49, 0.25)	64	−0.76 (−1.02, −0.50)***	−0.08 (−0.49, 0.32)
Sex						
Female	64	−0.45 (−0.69, −0.22)***	Reference	50	−0.68 (−0.93, −0.42)***	Reference
Mixed	53	−0.71 (−0.94, −0.47)***	−0.25 (−0.58, 0.07)	63	−0.77 (−1.01, −0.54)***	−0.10 (−0.44, 0.25)
Clinical severity						
No clinical symptoms	85	−0.40 (−0.57, −0.22)***	Reference	75	−0.47 (−0.64, −0.30)***	Reference
Clinical symptoms	34	−1.01 (−1.33, −0.69)***	−0.62 (−0.98, −0.26)**	36	−1.30 (−1.66, −0.95)***	−0.83 (−1.22, −0.44)***
Treatment duration						
Short duration (≤ 28 d)	27	−0.65 (−1.05, −0.25)**	Reference	34	−0.83 (−1.19, −0.47)***	Reference
Medium duration (29-56d)	66	−0.62 (−0.85, −0.40)***	0.03 (−0.42, 0.48)	53	−0.66 (−0.89, −0.43)***	0.17 (−0.25, 0.59)
Long duration (> 56 d)	19	−0.34 (−0.66, −0.01)*	0.32 (−0.17, 0.80)	14	−0.68 (−1.21, −0.15)*	0.15 (−0.47, 0.78)
Treatment frequency						
Low (< 1/w)	6	−0.19 (−0.83, 0.45)	0.43 (−0.24, 1.11)	8	−0.30 (−0.71, 0.11)	0.59 (0.09, 1.10)*
Medium (1/w)	46	−0.52 (−0.78, −0.26)***	0.10 (−0.25, 0.44)	35	−0.59 (−0.87, −0.30)***	0.31 (−0.08, 0.69)
High (> 1/w)	55	−0.62 (−0.85, −0.38)***	Reference	53	−0.89 (−1.16, −0.63)***	Reference
Total exposure						
Very short (≤ 4 h)	10	−0.40 (−0.80, −0.00)*	Reference	23	−0.52 (−0.79, 0.25)***	Reference
Low (4–12 h)	41	−0.51 (−0.80, −0.22)***	−0.11 (−0.59, 0.38)	31	−0.81 (−1.21, −0.41)***	−0.29 (−0.76, 0.18)
High (12.1–24 h)	32	−0.40 (−0.68, −0.12)**	−0.00 (−0.48, 0.48)	25	−0.55 (−0.80, −0.31)***	−0.04 (−0.39, 0.31)
Very high (> 24 h)	15	−1.19 (−1.74, −0.63)***	−0.78 (−1.44, −0.13)*	14	−1.44 (−2.09, −0.80)***	−0.93 (−1.59, −0.26)*
Format						
Group	64	−0.56 (−0.79, −0.32)***	Reference	50	−0.64 (−0.89, −0.38)***	Reference
Individual	45	−0.65 (−0.91, −0.38)***	−0.09 (−0.44, 0.26)	57	−0.88 (−1.14, −0.61)***	−0.24 (−0.61, 0.12)
Mixed	3	−0.15 (−0.51, 0.20)	0.40 (−0.17, 0.98)	4	−0.50 (−2.14, 1.13)	0.13 (−1.43, 1.69)
Supervision						
Supervised	94	−0.62 (−0.80, −0.43)***	Reference	83	−0.79 (−0.99, −0.59)***	Reference
Unsupervised	20	−0.46 (−0.88, −0.04)*	0.15 (−0.30, 0.61)	29	−0.65 (−1.08, −0.22)**	0.14 (−0.32, 0.61)
Location						
Face-to-face	100	−0.63 (−0.82, −0.44)***	Reference	97	−0.83 (−1.03, −0.63)***	Reference
Remote	13	−0.30 (−0.52, −0.09)*	0.33 (0.03, 0.62)*	14	−0.32 (−0.56, −0.08)	0.51 (0.19, 0.83)**
Home practice						
Present	58	−0.67 (−0.92, −0.43)***	Reference	40	−0.53 (−0.79, −0.28)***	Reference
Absent	51	−0.53 (−0.78, −0.27)***	0.15 (−0.20, 0.49)	61	−0.92 (−1.19, −0.66)***	−0.39 (−0.75, −0.03)*

### Network Meta-Analyses

**Fig. 2:**
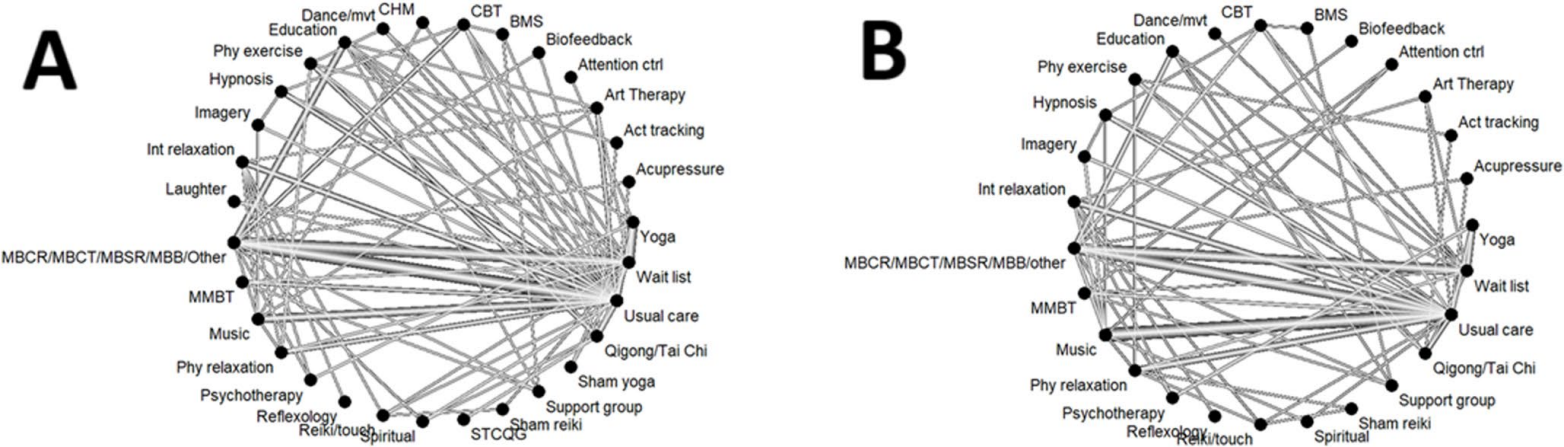
Network meta-analysis plot of the meta-analyses on depression symptoms (**A**) and anxiety symptoms (**B**). *BMS *body-mind-spirit intervention, *CBT* cognitive-behavioural therapy, *CHM* Chinese herbal medicine, *MBB* mind-body bridging, *MBCR* mindfulness-based cancer recovery, *MBCT* mindfulness-based cognitive therapy, *MBSR* mindfulness-based stress reduction therapy, *MMBT* multicomponent mind-body therapies, *TCQG* Tai Chi/qigong, *STCQG* sham qigong/Tai Chi, *VR* virtual reality

A total of 150, 138, and 182 studies were included in the network meta-analysis on depression symptoms, anxiety symptoms, and acceptability, respectively. The network meta-analysis plots for the meta-analyses on depression and anxiety symptoms are presented in Fig. 2.

All the MBTs had a larger effect on depression symptoms than usual care (Table 2). Four interventions, including biofeedback, mindfulness-based interventions (MBI), spiritual interventions, and dance/movement therapy, had a high treatment effect (SMD > 0.8) compared to usual care. The difference with usual care was statistically significant for MBIs, spiritual interventions, integrative relaxation, yoga, physical relaxation, and music therapy.

**Table 2:** Network meta-analysis of the depression symptoms in experimental and control interventions compared to usual care and SUCRA values

Intervention	Groups (participants)	Difference with usual care			SUCRA
		SMD (95% CI)	Z value	*p* value	
Biofeedback	2 (38)	−1.18 (−2.30, −0.06)	−2.07	0.038	0.83
MBIs	46 (2030)	−0.87 (−1.13, −0.61)	−6.66	< 0.001	0.78
Reflexology	1 (40)	−1.15 (−2.71, 0.42)	−1.43	0.152	0.77
Spiritual interventions	2 (29)	−0.98 (−1.95, −0.01)	−1.98	0.047	0.76
Dance/movement therapy	2 (82)	−0.83 (−1.86, 0.21)	−1.57	0.116	0.68
Imagery/visualisation	7 (165)	−0.75 (−1.56, 0.06)	−1.81	0.071	0.66
Integrative relaxation	8 (310)	−0.71 (−1.23, −0.20)	−2.74	0.006	0.66
Laughter therapy	2 (39)	−0.76 (−1.86, 0.35)	−1.34	0.179	0.65
Yoga	24 (692)	−0.68 (−1.08, −0.27)	−3.29	0.001	0.64
Art therapy	4 (142)	−0.64 (−1.30, 0.02)	−1.90	0.058	0.60
Physical relaxation	8 (243)	−0.59 (−1.11, −0.07)	−2.22	0.026	0.58
Education	11 (462)	−0.58 (−1.06, −0.10)	−2.37	0.018	0.58
Sham qigong/Tai Chi	1 (45)	−0.60 (−1.74, 0.55)	−1.02	0.306	0.57
Music therapy	18 (692)	−0.56 (−0.95, −0.16)	−2.78	0.006	0.56
Cognitive-behavioural therapy	5 (222)	−0.54 (−1.16, 0.08)	−1.71	0.088	0.53
Body-mind-spirit intervention	2 (105)	−0.49 (−1.44, 0.47)	−1.00	0.319	0.51
Activity tracking	1 (8)	−0.48 (−2.05, 1.08)	−0.60	0.546	0.50
Support group	5 (258)	−0.40 (−1.29, 0.48)	−0.89	0.373	0.46
Acupressure	1 (36)	−0.37 (−1.67, 0.93)	−0.56	0.575	0.45
Attention control	1 (19)	−0.24 (−1.84, 1.36)	−0.29	0.771	0.41
Chinese herbal medicine	1 (37)	−0.30 (−1.60, 1.00)	−0.46	0.648	0.40
Reiki/touch therapy	2 (41)	−0.29 (−1.33, 0.75)	−0.54	0.590	0.38
Exercise	7 (208)	−0.32 (−0.98, 0.35)	−0.94	0.349	0.38
Multicomponent mind-body-therapy	6 (495)	−0.28 (−0.90, 0.34)	−0.88	0.379	0.37
Qigong/Tai Chi	12 (432)	−0.31 (−0.77, 0.15)	−1.31	0.191	0.37
Waitlist control	47 (1570)	−0.30 (−0.62, 0.02)	−1.87	0.062	0.36
Sham reiki/touch therapy	1 (27)	−0.10 (−1.53, 1.34)	−0.13	0.896	0.35
Hypnotherapy	6 (157)	−0.20 (−0.84, 0.44)	−0.61	0.542	0.34
Usual care	68 (2641)	Reference			0.17
Sham yoga	1 (52)	0.45 (−0.87, 1.76)	0.66	0.508	0.14
Psychotherapy	3 (91)	0.42 (−0.39, 1.24)	1.02	0.309	0.09

All the MBTs had a larger effect on anxiety symptoms than usual care (Table 3). Seven interventions, including yoga, MBIs, physical relaxation, spiritual interventions, music therapy, qigong/Tai Chi, and biofeedback, had a high treatment effect (SMD > 0.8) compared to usual care. The difference with usual care was statistically significant for yoga, MBIs, physical relaxation, music therapy, qigong/Tai Chi, imagery/visualisation, and integrative relaxation.

**Table 3:** Network meta-analysis of the anxiety symptoms in experimental and control interventions compared to usual care with SUCRA values

Intervention	Groups (participants)	Difference with usual care			SUCRA
		SMD (95% CI)	Z value	*p* value	
Yoga	16 (450)	−1.13 (−1.60, −0.67)	−4.76	< 0.001	0.80
MBIs	42 (1,781)	−1.02 (−1.30, −0.74)	−7.08	< 0.001	0.75
Physical relaxation	13 (330)	−0.93 (−1.38, −0.47)	−4.01	< 0.001	0.68
Spiritual interventions	1 (53)	−0.99 (−2.27, 0.30)	−1.50	0.133	0.66
Music therapy	32 (1,109)	−0.90 (−1.17, −0.62)	−6.40	< 0.001	0.65
Qigong/Tai Chi	6 (213)	−0.90 (−1.51, −0.29)	−2.88	0.004	0.64
Activity tracking	1 (8)	−0.99 (−2.62, 0.65)	−1.18	0.237	0.63
Biofeedback	1 (21)	−0.95 (−2.53, 0.63)	−1.18	0.237	0.59
Cognitive-behavioural therapy	5 (199)	−0.80 (−1.50, −0.10)	−2.25	0.024	0.59
Art therapy	1 (20)	−0.76 (−1.80, 0.28)	−1.44	0.151	0.56
Reflexology	1 (40)	−0.80 (−2.37, 0.77)	−1.00	0.317	0.55
Body-mind-spirit intervention	2 (105)	−0.77 (−1.98, 0.45)	−1.23	0.218	0.55
Sham reiki/touch therapy	1 (19)	−0.76 (−2.24, 0.72)	−1.00	0.317	0.53
Imagery/visualisation	6 (114)	−0.72 (−1.42, −0.02)	−2.02	0.044	0.52
Integrative relaxation	13 (411)	−0.69 (−1.17, −0.21)	−2.81	0.005	0.49
Education	6 (228)	−0.64 (−1.28, 0.01)	−1.94	0.053	0.46
Multicomponent mind-body-therapy	6 (491)	−0.61 (−1.27, 0.05)	−1.81	0.071	0.46
Support group	4 (242)	−0.57 (−1.51, 0.37)	−1.19	0.232	0.45
Acupressure	1 (36)	−0.54 (−1.85, 0.77)	−0.81	0.418	0.44
Waitlist	34 (1,075)	−0.60 (−0.96, −0.23)	−3.21	0.001	0.42
Hypnotherapy	7 (167)	−0.55 (−1.13, 0.03)	−1.84	0.065	0.40
Reiki/touch therapy	2 (37)	−0.44 (−1.50, 0.61)	−0.82	0.411	0.38
Exercise	4 (77)	−0.47 (−1.40, 0.46)	−0.98	0.325	0.37
Dance/movement therapy	1 (64)	−0.42 (−1.93, 1.09)	−0.55	0.586	0.37
Attention control	2 (69)	−0.31 (−1.49, 0.87)	−0.51	0.608	0.32
Psychotherapy	5 (200)	0.06 (−0.69, 0.83)	0.17	0.864	0.12
Usual care	77 (2,799)	Reference			

The risk ratios of completion rate between MBTs and usual care were similar, ranging between RR = 0.94 and RR = 1.06, except Johri which had a risk ratio of RR = 0.58 (95% CI = 0.26 to 1.27) and laughter therapy which had a risk ratio of RR = 0.85 (95% CI = 0.72 to 1.01). The details of the network meta-analysis on acceptability are presented in Online Resource 6.

### Consistency Assessment

The direct and indirect estimates of effect for the depression, anxiety and acceptability network meta-analyses were compared. The assessment shows evidence of inconsistency in the estimates of effect for depression symptoms in 4 of the 71 meta-analyses, involving imagery/visualisation and, integrative relaxation. There is evidence of inconsistency in the estimates of effect for anxiety symptoms in 3 of the 55 pairwise comparisons between direct and indirect estimates of effect size, involving music, physical relaxation, and yoga. The global consistency assessment showed no evidence of global inconsistency for depression (Q = 65.44, *p* = 0.099) and anxiety symptoms (Q = 38.49, *p* = 0.538).

### Subgroup Analysis of the Network Meta-Analysis

In the subgroup analysis, the MBIs group was separated into MBCT, MBSR, and MBCR, music therapy into music engagement (i.e., active participation of participants) and music listening, and visualisation/imagery with or without virtual reality were separated. The subgroup analysis of depression symptoms shows that imagery/visualisation with virtual reality (SMD = −2.93) is more effective than imagery/visualisation without virtual reality (SMD = −0.05). Music listening (SMD = −0.83) was more effective than music engagement (SMD = 0.03). The effects of MBCR (SMD = −1.27) and other MBIs (SMD = −1.15) relative to usual care were larger than the effects of MBSR (SMD = −0.78) and MBCT (SMD = −0.72).

### Analysis of IPD

The IPD was available from 8 studies [60–67] for depression symptoms and from 6 studies [60–63, 66, 68] for anxiety symptoms. The main reasons for unavailability of IPD were authors not contactable (141 studies) and authors refused to provide data due to ethical concerns (15 studies) and due to a lack of resources (17 studies). After correcting for any baseline differences in depression scores and allowing clustering of baseline and end of treatment depression scores within studies, the inactive control group recorded a mean standardised score at follow-up which is a statistically significant 0.53 and 0.64 standard deviations higher than those in the MBT treatment group (*p* < 0.001 for both) for depression and anxiety symptoms, respectively.

## Discussion

### Main Findings

This is the first study to rank the effectiveness of a large pool of MBTs for depression and anxiety symptoms in people with cancer using an NMA. MBTs that produced a statistically significant large effect compared with usual care are biofeedback (SMD = −1.18), spiritual interventions (SMD = −0.98), and MBIs (SMD = −0.87) for depression symptoms and yoga (SMD = −1.13), MBIs (SMD = −1.02), physical relaxation (SMD = −0.93), qigong/Tai Chi (SMD = −0.90) and music therapy (SMD = −0.90) for anxiety symptoms. The relatively low effectiveness of multicomponent MBTs, which include non-MBT components such as education, diet therapy, and exercise suggested that including more components to the intervention may not increase effectiveness. The effect sizes for acceptability were small and not statistically (except for MBIs) nor clinically significant.

The subgroup analyses and meta-regressions showed that MBTs with a low session frequency (< 1/w) had a lower effect relative to control interventions than other MBTs, while interventions with a very high total exposure (> 24 h) had a higher effect, suggesting that a certain treatment dosage may be required for optimal effectiveness. Interestingly, the relative effect of MBTs with a long duration compared to control interventions was lower than other MBTs (especially for depression). One reason might be that MBTs ‘lose’ their effectiveness over time. The lower relative effect of remote interventions is consistent with the literature on different formats of behavioural interventions [69, 70]. MBT protocols using an individual format and supervision had a slightly higher (but not statistically different) effect compared with protocols using a group format and without supervision. This slightly higher effect of the group format of behavioural interventions for mood impairment is consistent with the literature [71].

The subgroup analyses showed a lower effect for people with breast cancer, early-stage cancer and females, which is paradoxically the population most prone to use MBTs [72] and on which clinical research is focused [73]. These findings suggested that the best potential of MBT may not be utilised in the clinic and that clinical research may underestimate the effect of MBTs. The effect of MBT was higher in the studies in which the study sample reached, on average, the questionnaire thresholds for clinical anxiety and depression.

In this study, we found that MBT had an overall effect of SMD = −0.58 on depression symptoms and SMD = −0.74 on anxiety symptoms compared to inactive control interventions. Several MBTs had an effect superior to 1.00 compared to usual care. These effects are relatively high, especially considering the effect of the recommended interventions for anxiety and depression symptoms, CBT, antidepressants and anxiolytics [74]. Indeed, previous studies show that CBT had an effect of SMD = −0.88 to −0.83 for depressive symptoms [75, 76] and SMD = −0.97 (pre-treatment versus post-treatment) and SMD = −0.55 (between CBT and control groups) for anxiety symptoms, antidepressant medications had an effect of SMD = −0.60 to −0.23 for depressive symptoms [75, 77, 78], and benzodiazepine medications had an effect of SMD = −0.58 for anxiety symptoms [79]. However, the promising effect of MBT should be considered with caution due to the potential risk of bias identified in most of the studies and the potential publication bias.

### Study Limitations

The largest limitation of this study is that it did not conduct the network meta-analysis with IPD as was originally planned in the protocol due to lack of data availability. As a result, we are unable to rank the effect of MBTs in people with cancer with clinically significant levels of depression. This is an important point as the subgroup analysis of our study suggested that patients with clinical depression may benefit more from MBT than patients without clinical depression. The completion rate is a relatively accessible measure, however, it may not reflect all the aspects of intervention acceptability, which is a complex topic [77]. Finally, the evidence of inconsistency suggests that some factors such as population characteristics and intervention format may have impacted the effectiveness of the interventions. The results of this meta-analysis should therefore be interpreted with caution.

### Clinical Implications

Our study addresses the lack of comparison in the literature between the relative effects of MBTs for depression and anxiety symptoms in people with cancer and provides guidance to clinicians about the best interventions for these symptoms. The results of this study suggests that clinicians and health care decision-makers should prioritise (in the following order) biofeedback, spiritual interventions and MBIs for depression symptoms and yoga, MBIs, physical relaxation, qigong/Tai Chi and music therapy for anxiety symptoms when they are considering mind-body therapies for the management of depression and anxiety symptoms in people with cancer. The results also suggests that MBT with a low session frequency, a long intervention duration (for depression only) and a remote format should be avoided as they may be less effective. The use of MBT for depression and anxiety symptoms should not be limited to female with breast cancer as other cancer populations may receive similar or superior benefit from MBT.

## Conclusion

In conclusion, the most effective MBTs are biofeedback, spiritual interventions and MBIs for depression symptoms and yoga, MBIs, physical relaxation, qigong/Tai Chi and music therapy for anxiety symptoms in people with cancer. More research is required for biofeedback, spiritual interventions and dance/movement therapy which are promising yet supported by too few studies. We recommend to clinicians to avoid MBT interventions with a low session frequency, a very short total exposure, and a long duration, and remote interventions.

## Supplementary Information

Below is the link to the electronic supplementary material.

Supplementary Material 1 (DOCX 16.8 KB)

Supplementary Material 2 (DOCX 23.3 KB)

Supplementary Material 3 (DOCX 17.8 KB)

Supplementary Material 4 (DOCX 59.5 KB)

Supplementary Material 5 (DOCX 216 KB)

Supplementary Material 6 (DOCX 78.0 KB)

## Data Availability

The data underlying this article are available in figshare at https://dx.doi.org/10.6084/m9.figshare.24902124.
