# Supplementary material for: Mind-Body Therapies for Depression and Anxiety Symptoms in People with Cancer: A Systematic Review with Network Meta-Analysis
Source: Curr Oncol Rep. 2026 May 18;28(1):52. doi: 10.1007/s11912-026-01790-7 (PMC13183694; doi:10.1007/s11912-026-01790-7)
Supplement: Supplementary file 1 — Supplementary Material 1 (DOCX 16.8 KB) [file 11912_2026_1790_MOESM1_ESM.docx]

# Supplementary Material 1 – List of acceptable questionnaires

Article title: Mind-body therapies for depression and anxiety symptoms in people with cancer: A systematic review with network meta-analysis

Journal name: Current Oncology Reports

Authors: Yoann Birling, Deep J. Bhuyan, Fan Feng, Jing Liu, Linda E. Carlson, Mingxian Jia, Wing Yu Yu, Han Zhang, Matthew Rahimi, Nibras Jasim, Betul H. Boge, Sarah Nevitt, Kayla Jaye, Indeewarie D. Mudiyanselage, Changrong Tang, Tiffany Tram, Judith Lacey, Rogier Hoenders, Paul P. Fahey.

Corresponding author: Yoann Birling, NICM Health Research Institute, Western Sydney University, yoannbirling@gmail.com.

This supplementary material presents the questionnaires that were considered acceptable for inclusion in the systematic review.

The self-reported questionnaires assessing depression symptoms accepted in this review included

- Patient Health Questionnaire (PHQ-9)
- Center for Epidemiological Studies Depression (CES-D)
- Beck Depression Index (BDI)
- Hamilton Depression Rating Scale (HAMD)
- Profile of Mood State – Depression (POMS-D)
- Hospital Anxiety and Depression Scale – Depression (HADS-D)
- Patient-Reported Outcomes Measurement Information System - Depression (PROMIS-D)
- Depression, Anxiety and Stress Scale – Depression (DASS-D)
- Symptoms Checklist 90-items (SCL-90)
- Montgomery–Åsberg Depression Rating Scale (MADRS)

The self-reported questionnaires assessing depression symptoms accepted in this review included

- Beck Anxiety Index (BAI)
- Depression Anxiety and Stress Scale – Anxiety subscale (DASS-A)
- Generalised Anxiety Disorder – 7 items (GAD-7)
- Generalised Anxiety Disorder – 2 items (GAD-2)
- Hospital Anxiety and Depression Scale – Anxiety subscale (HADS-A)
- Hamilton Anxiety Rating Scale (HAMA)
- Profile of Mood States – Anxiety subscale (POMS-A)
- Patient-Reported Outcomes Measurement Information System – Anxiety subscale (PROMIS-A)
- State-Trait Anxiety Scale (STAI)
- State-Trait Anxiety Scale – State subscale (STAI-S)
- Penn State Worry Questionnaire (PSWQ)
