# Supplementary material for: Mind-Body Therapies for Depression and Anxiety Symptoms in People with Cancer: A Systematic Review with Network Meta-Analysis
Source: Curr Oncol Rep. 2026 May 18;28(1):52. doi: 10.1007/s11912-026-01790-7 (PMC13183694; doi:10.1007/s11912-026-01790-7)
Supplement: Supplementary file 2 — Supplementary Material 2 (DOCX 23.3 KB) [file 11912_2026_1790_MOESM2_ESM.docx]

# Supplementary Material 2 - Search strategy and list of systematic reviews

Article title: Mind-body therapies for depression and anxiety symptoms in people with cancer: A systematic review with network meta-analysis

Journal name: Current Oncology Reports

Authors: Yoann Birling, Deep J. Bhuyan, Fan Feng, Jing Liu, Linda E. Carlson, Mingxian Jia, Wing Yu Yu, Han Zhang, Matthew Rahimi, Nibras Jasim, Betul H. Boge, Sarah Nevitt, Kayla Jaye, Indeewarie D. Mudiyanselage, Changrong Tang, Tiffany Tram, Judith Lacey, Rogier Hoenders, Paul P. Fahey.

Corresponding author: Yoann Birling, NICM Health Research Institute, Western Sydney University, yoannbirling@gmail.com.

This supplementary material presents the search strategy used for each of the databases searched during this review and the list of the systematic reviews that were hand-searched.

## Search strategy

Below is the search strategy for PubMed. This search strategy was adapted for the other databases.

PubMed search (with the filters “Clinical Trial” and “Randomized Controlled Trial” applied)

| #1 | "mind-body therapies"[MeSH Terms] OR "mind body therap*"[Title/Abstract] OR "yoga"[MeSH Terms] OR "qigong"[MeSH Terms] OR "tai ji"[MeSH Terms] OR "mindfulness"[MeSH Terms] OR "meditation"[MeSH Terms] OR "yoga"[Title/Abstract] OR "qigong"[Title/Abstract] OR "qi gong"[Title/Abstract] OR "taiji"[Title/Abstract] OR "taichi"[Title/Abstract] OR "tai ji"[Title/Abstract] OR "tai chi"[Title/Abstract] OR "mindfulness"[Title/Abstract] OR "relaxation"[Title/Abstract] OR "meditation"[Title/Abstract] |
| --- | --- |
| #2 | "neoplasms"[MeSH Terms] OR "cancer survivors"[MeSH Terms] OR "neoplasms"[Title/Abstract] OR "tumor"[Title/Abstract] OR "cancer"[Title/Abstract] OR "leukemia"[Title/Abstract] OR "myeloma"[Title/Abstract] OR "lymphoma"[Title/Abstract] |
| #3 | "sleep initiation and maintenance disorders"[MeSH Terms] OR "insomnia"[Title/Abstract] OR "sleeplessness"[Title/Abstract] OR "sleep disturbances"[Title/Abstract] OR "sleep"[Title/Abstract] OR "depressive disorder"[MeSH Terms] OR "depression"[MeSH Terms] OR "depressive disorder"[MeSH Terms] OR "depressive disorder, major"[MeSH Terms] OR "dysthymic disorder"[MeSH Terms] OR "adjustment disorders"[MeSH Terms] OR "depression"[Title/Abstract] OR "mood"[Title/Abstract] OR "anxiety"[MeSH Terms] OR "anxiety"[Title/Abstract] OR "anxiety disorders"[Title/Abstract] OR "phobic disorders"[MeSH Terms] OR "mental health"[MeSH Terms] OR "mental disorders"[MeSH Terms] OR "psycholog*"[Title/Abstract] |
| #4 | #1 AND #2 AND #3 |

## List of the systematic reviews searched manually

Below is the list of the 111 systematic reviews on the topic of mind-body therapies in cancer patients that were searched manually.

Abbott-Anderson, K. and K. L. Kwekkeboom. 2021. A systematic review of sexual concerns reported by gynecological cancer survivors

Araujo, R. V., et al. 2019. Meditation effect on psychological stress level in women with breast cancer: a systematic review

Archer, S., et al. 2015. The effect of creative psychological interventions on psychological outcomes for adult cancer patients: a systematic review of randomised controlled trials

Bergenthal, N., et al. 2014. Aerobic physical exercise for adult patients with haematological malignancies.

Billones, R. and L. Saligan. 2020. What Works in Mindfulness Interventions for Medically Unexplained Symptoms? A Systematic Review

Boehm, K., et al. 2014. Arts therapies for anxiety, depression, and quality of life in breast cancer patients: a systematic review and meta-analysis

Bradt, J., et al. Music interventions for improving psychological and physical outcomes in cancer patients. 2011. Music interventions for improving psychological and physical outcomes in cancer patients

Bradt, J., et al. 2016. Music interventions for improving psychological and physical outcomes in cancer patients

Bradt, J., et al. Dance/movement therapy for improving psychological and physical outcomes in cancer patients. 2011. Dance/movement therapy for improving psychological and physical outcomes in cancer patients

Bradt, J., et al. 2015. Dance/movement therapy for improving psychological and physical outcomes in cancer patients

Brighton, L. J., et al. 2019. Holistic services for people with advanced disease and chronic breathlessness: a systematic review and meta-analysis

Bro, M. L., et al. 2018. Kind of blue: A systematic review and meta-analysis of music interventions in cancer treatment

Casellas-Grau, A., et al. Positive psychology interventions in breast cancer. A systematic review. 2014. Positive psychology interventions in breast cancer. A systematic review.

Chen, D., et al. 2018. Fear of Cancer Recurrence: A Systematic Review of Randomized, Controlled Trials

Cillessen, L., et al. 2019. Mindfulness-based interventions for psychological and physical health outcomes in cancer patients and survivors: A systematic review and meta-analysis of randomized controlled trials

Coughtrey, A., et al. The Effectiveness of Psychosocial Interventions for Psychological Outcomes in Pediatric Oncology: A Systematic Review. 2018. The Effectiveness of Psychosocial Interventions for Psychological Outcomes in Pediatric Oncology: A Systematic Review.

Coutiño-Escamilla, L., et al. 2019. Intervenciones no farmacológicas para reducir síntomas depresivos en mujeres con cáncer de mam [Non-pharmacological interventions to reduce depressive symptoms in women with breast cancer]

Cramer, H., et al. 2017. Yoga for improving health-related quality of life, mental health and cancer-related symptoms in women diagnosed with breast cancer.

Cramer, H., et al. 2012. Mindfulness-based stress reduction for breast cancer-a systematic review and meta-analysis

D'Silva, S., et al. Mind-body medicine therapies for a range of depression severity: a systematic review. 2012. Mind-body medicine therapies for a range of depression severity: a systematic review

Dalton, J., et al. 2018. Updated meta-review of evidence on support for carers.

Dharmawardene, M., et al. 2016. A systematic review and meta-analysis of meditative interventions for informal caregivers and health professionals.

Du, S., et al. 2015. Patient education programs for cancer-related fatigue: A systematic review

Estrella-Castillo, D., et al. 2020. Scientific evidence of the relation between breast cancer and depression: systematic review

Faller, H., et al. 2013. Effects of psycho-oncologic interventions on emotional distress and quality of life in adult patients with cancer: systematic review and meta-analysis

Flowers, S. R. and K. A. Birnie. 2015. Procedural Preparation and Support as a Standard of Care in Pediatric Oncology

Fu, F., et al. 2017. A Systematic Review of Psychosocial Interventions to Cancer Caregivers

Galaal, K., et al. 2011. Interventions for reducing anxiety in women undergoing colposcopy

Galaal, K. A., et al. 2007. Interventions for reducing anxiety in women undergoing colposcopy

Galliford, M., et al. 2017. Salute to the sun: a new dawn in yoga therapy for breast cancer.

Gotink, R. A., et al. 2015. Standardised mindfulness-based interventions in healthcare: an overview of systematic reviews and meta-analyses of RCTs.

Greenlee, H., et al. 2014. Clinical practice guidelines on the use of integrative therapies as supportive care in patients treated for breast cancer

Greenlee, H., et al. 2017. Clinical practice guidelines on the evidence-based use of integrative therapies during and after breast cancer treatment

Haller, H., et al. 2017. Mindfulness-based interventions for women with breast cancer: an updated systematic review and meta-analysis

Henshall, C. L., et al. 2019. A Systematic Review and Narrative Synthesis to Explore the Effectiveness of Exercise-Based Interventions in Improving Fatigue, Dyspnea, and Depression in Lung Cancer Survivors

Hersch, J., et al. 2009. Psychosocial interventions and quality of life in gynaecological cancer patients: a systematic review.

Hetkamp, M., et al. 2019. A Systematic Review of the Effect of Neurofeedback in Cancer Patients

Hulett, J. M. and J. M. Armer. 2016. A Systematic Review of Spiritually Based Interventions and Psychoneuroimmunological Outcomes in Breast Cancer Survivorship

Huston, P. and B. McFarlane. 2016. Health benefits of tai chi: What is the evidence?

Jain, S. and P. J. Mills. 2010. Biofield therapies: helpful or full of hype? A best evidence synthesis.

Kapogiannis, A., et al. 2018. Investigating the Effects of the Progressive Muscle Relaxation-Guided Imagery Combination on Patients with Cancer Receiving Chemotherapy Treatment: A Systematic Review of Randomized Controlled Trials

Kawashima, Y., et al. 2019. Interventions to prevent suicidal behavior and ideation for patients with cancer: A systematic review

Klein, P. J., et al. 2019. Qigong and Tai Chi as Therapeutic Exercise: Survey of Systematic Reviews and Meta-Analyses Addressing Physical Health Conditions.

Knips, L., et al. 2019. Aerobic physical exercise for adult patients with haematological malignancies

Köhler, F., et al. 2020. Music Therapy in the Psychosocial Treatment of Adult Cancer Patients: A Systematic Review and Meta-Analysis

Kreutz, C., et al. 2019. Effects of physical and mind-body exercise on sleep problems during and after breast cancer treatment: a systematic review and meta-analysis

Kühne, F., et al. 2016. Psychological treatments for pain in cancer patients : A systematic review on the current state of research

Li, Y., et al. 2020. The effectiveness of music therapy for patients with cancer: A systematic review and meta-analysis

Liu, L., et al. 2020. The effectiveness of tai chi in breast cancer patients: A systematic review and meta-analysis

Luo, X. C., et al. 2020. Effect of Tai Chi Chuan in Breast Cancer Patients: A Systematic Review and Meta-Analysis

Machado, F. V., et al. 2020. More than a quarter century of the most prescribed sleeping pill: Systematic review of zolpidem use by older adult

Matthews, E., et al. 2018. Sleep-Wake Disturbance: A Systematic Review of Evidence-Based Interventions for Management in Patients With Cancer

Matthews, E. E., et al. 2018. Effects of Exercise on Sleep in Women with Breast Cancer: A Systematic Review

McKay, F. H., et al. 2018. Evaluating mobile phone applications for health behaviour change: A systematic review

Medisauskaite, A. and C. Kamau. 2017. Prevalence of oncologists in distress: Systematic review and meta-analysis

Mishra, S. I., et al. 2012. Exercise interventions on health-related quality of life for cancer survivors.

Mishra, S. I., et al. 2012. Exercise interventions on health-related quality of life for people with cancer during active treatment

Montgomery, G. H., et al. 2017. Hypnosis for Symptom Control in Cancer Patients at the End-of-Life: A Systematic Review.

Moore, D. A., et al. 2019. Interventions to improve the mental health of children and young people with long-term physical conditions: linked evidence syntheses

Moore, T. H., et al. 2015. Supportive care for men with prostate cancer: why are the trials not working? A systematic review and recommendations for future trials.

Moraes, L. J., et al. 2018. A systematic review of psychoneuroimmunology-based interventions

Mosher, C. E., et al. 2017. A systematic review of psychosocial interventions for colorectal cancer patients

Musial, F., et al. 2011. Mindfulness-based stress reduction for integrative cancer care: a summary of evidence

Ngamkham, S., et al. 2019. A Systematic Review: Mindfulness Intervention for Cancer-Related Pain

Ni, X., et al. 2019. The effects of Tai Chi on quality of life of cancer survivors: a systematic review and meta-analysis

Nightingale, C. L., et al. 2013. The impact of music interventions on anxiety for adult cancer patients: a meta-analysis and systematic review

Nunns, M., et al. 2018. Effectiveness of nonpharmacological interventions to reduce procedural anxiety in children and adolescents undergoing treatment for cancer: A systematic review and meta-analysis

Nzwalo, I., et al. 2020. Systematic Review of the Prevalence, Predictors, and Treatment of Insomnia in Palliative Care

Oberoi, S., et al. 2020. Association of Mindfulness-Based Interventions With Anxiety Severity in Adults With Cancer: A Systematic Review and Meta-analysis

Panchik, D., et al. 2019. Effect of Exercise on Breast Cancer-Related Lymphedema: What the Lymphatic Surgeon Needs to Know

Parikh, M., et al. 2019. Characteristics and Components of Medical Group Visits for Chronic Health Conditions: A Systematic Scoping Review

Pelekasis, P., et al. 2017. Progressive muscle relaxation as a supportive intervention for cancer patients undergoing chemotherapy: A systematic review

Pene, C. T. H. and D. Kissane. 2019. Communication in cancer: its impact on the experience of cancer care: communicating with the angry patient and the patient in denial

Petricone-Westwood, D., et al. 2019. A Systematic Review of Interventions for Health Anxiety Presentations Across Diverse Chronic Illnesses.

Piet, J., et al. 2012. The effect of mindfulness-based therapy on symptoms of anxiety and depression in adult cancer patients and survivors: a systematic review and meta-analysis.

Rajasekaran, M., et al. 2005. Systematic review of hypnotherapy for treating symptoms in terminally ill adult cancer patients

Richardson, A. E., et al. 2019. A systematic review of psychological interventions for patients with head and neck cancer

Richardson, J., et al. 2006. Hypnosis for procedure-related pain and distress in pediatric cancer patients: a systematic review of effectiveness and methodology related to hypnosis interventions.

Rodin, G., et al. 2007. The treatment of depression in cancer patients: a systematic review

Roffe, L., et al. 2005. A systematic review of guided imagery as an adjuvant cancer therapy

Rush, S. E. and M. Sharma. 2017. Mindfulness-Based Stress Reduction as a Stress Management Intervention for Cancer Care: A Systematic Review

Salhofer, I., et al. 2016. Meditation for adults with haematological malignancies.

Sanjida, S., et al. 2018. Are psychological interventions effective on anxiety in cancer patients? A systematic review and meta-analyses.

Sasaki, Y., et al. 2019. Complementary and Alternative Medicine for Breast Cancer Patients: An Overview of Systematic Reviews

Schell, L. K., et al. 2019. Mindfulness-based stress reduction for women diagnosed with breast cancer

Shennan, C., et al. 2011. What is the evidence for the use of mindfulness-based interventions in cancer care? A review

Smith, J. E., et al. 2005. Mindfulness-Based Stress Reduction as supportive therapy in cancer care: systematic review

Stanhope, J. and P. Weinstein. 2020. The human health effects of singing bowls: A systematic review

Tamagawa, R., et al. 2012. Who benefits from psychosocial interventions in oncology? A systematic review of psychological moderators of treatment outcome

Tang, M., et al. 2020. The Effects of Cognitive-Behavioral Stress Management for Breast Cancer Patients: A Systematic Review and Meta-analysis of Randomized Controlled Trials.

Tao, W., et al. 2015. Practice of traditional Chinese medicine for psycho-behavioral intervention improves quality of life in cancer patients: A systematic review and meta-analysis

Tay, L. H., et al. 2010. A systematic review on the factors affecting effective communication between registered nurses and oncology adult patients in an inpatient setting

Thrane, S. 2013. Effectiveness of integrative modalities for pain and anxiety in children and adolescents with cancer: a systematic review

Toneti, B. F., et al. 2020. Benefits of Qigong as an integrative and complementary practice for health: a systematic review

Treanor, C. J., et al. 2016. Non-pharmacological interventions for cognitive impairment due to systemic cancer treatment

van der Wal-Huisman, H., et al. 2018. The effect of music on postoperative recovery in older patients: A systematic review

Vira, P., et al. 2021. Role of Physiotherapy in Hospice Care of Patients with Advanced Cancer: A Systematic Review

Visovsky, C. and C. Dvorak. 2005. Exercise and cancer recovery

Wang, X., et al. 2018. Effects of Music Intervention on the Physical and Mental Status of Patients with Breast Cancer: A Systematic Review and Meta-Analysis

Wayne, P. M., et al. 2018. Tai Chi and Qigong for cancer-related symptoms and quality of life: a systematic review and meta-analysis

Wu, C., et al. 2019. Nonpharmacological Interventions for Cancer-Related Fatigue: A Systematic Review and Bayesian Network Meta-Analysis

Xiang, Y., et al. 2017. Does Tai Chi relieve fatigue? A systematic review and meta-analysis of randomized controlled trials.

Xunlin, N. G., et al. 2020. The effectiveness of mindfulness-based interventions among cancer patients and survivors: a systematic review and meta-analysis.

Yang, G. Y., et al. 2015. Evidence base of clinical studies on Tai Chi: a bibliometric analysis.

Zeng, Y., et al. 2014. Health benefits of qigong or tai chi for cancer patients: a systematic review and meta-analyses.

Zeng, Y., et al. 2019. Qigong or Tai Chi in Cancer Care: an Updated Systematic Review and Meta-analysis.

Zhang, J., et al. 2016. Effects of mindfulness-based therapy for patients with breast cancer: A systematic review and meta-analysis

Zhang, J. M., et al. 2012. Music interventions for psychological and physical outcomes in cancer: a systematic review and meta-analysis

Zhang, Q., et al. 2019. Effectiveness of mindfulness-based stress reduction (MBSR) on symptom variables and health-related quality of life in breast cancer patients-a systematic review and meta-analysis.

Zimmermann, F. F., et al. 2018. The acceptability and potential benefits of mindfulness-based interventions in improving psychological well-being for adults with advanced cancer: A systematic review
