# Supplementary material for: Mind-Body Therapies for Depression and Anxiety Symptoms in People with Cancer: A Systematic Review with Network Meta-Analysis
Source: Curr Oncol Rep. 2026 May 18;28(1):52. doi: 10.1007/s11912-026-01790-7 (PMC13183694; doi:10.1007/s11912-026-01790-7)
Supplement: Supplementary file 3 — Supplementary Material 3 (DOCX 17.8 KB) [file 11912_2026_1790_MOESM3_ESM.docx]

# Supplementary Material 3 - Grouping of MBT interventions

Article title: Mind-body therapies for depression and anxiety symptoms in people with cancer: A systematic review with network meta-analysis

Journal name: Current Oncology Reports

Authors: Yoann Birling, Deep J. Bhuyan, Fan Feng, Jing Liu, Linda E. Carlson, Mingxian Jia, Wing Yu Yu, Han Zhang, Matthew Rahimi, Nibras Jasim, Betul H. Boge, Sarah Nevitt, Kayla Jaye, Indeewarie D. Mudiyanselage, Changrong Tang, Tiffany Tram, Judith Lacey, Rogier Hoenders, Paul P. Fahey.

Corresponding author: Yoann Birling, NICM Health Research Institute, Western Sydney University, [yoannbirling@gmail.com](mailto:yoannbirling@gmail.com).

This supplementary presents the interventions that were grouped under the name of interventions as seen in the manuscript.

Art therapy included

- Art therapy
- Calligraphy
- Poetry
- Mindfulness-based art therapy
- Creative art therapy

Biofeedback included

- Biofeedback relaxation
- HRV biofeedback training

Dance/movement therapy included

- Belly dance training
- Dance/movement therapy

Hypnotherapy included

- Hypnosis
- Hypnotic recording
- Self-hypnosis

Integrative relaxation included any combination of the following

- Music relaxation (including progressive muscular relaxation)
- Physical relaxation (muscular and breathing)
- Visualization/imagery

Other Mindfulness-Based Interventions included

- Mindfulness meditation
- Flow meditation
- Mind substraction meditation
- Mindful awareness practice
- Couple-based meditation
- Tibetan sound meditation
- Body scan
- Mindfulness training
- Thai-Buddhism based meditation
- Mind-body bridging

Multicomponent MBT included any combination of MBT with

- Education
- Diet therapy
- Psychotherapy
- Exercise

Music therapy included

- Music listening
- Five element music
- Five tone therapy
- Traditional music
- Music engagement (producing music)
- Singing

Physical relaxation included

- Muscular relaxation
- Breathing relaxation
- A combination of muscular and breathing relaxation

Qigong/Tai Chi included (regardless of presence or absence of music)

- Qigong
- Baduanjin
- Guolin qigong
- Tai Chi
- Tai Chi Chi
- A combination or mix of Qigong and Tai Chi

Reiki/touch therapy included

- Reiki
- Touch therapy

Spiritual interventions included

- Listening to Quran
- Spiritual meditation
- Intercessory Christian prayer

Visualisation/imagery included

- Guided imagery
- Problem-focused visualisation
- Relaxation through guided imagery

Yoga included

- Postures
- Pranayama
- Any combination of postures, pranayama and other components labelled ‘yoga’, including Tibetan yoga, Hatha yoga, Lyengar yoga, YOCAS, and dyads yoga
