# Supplementary material for: Mind-Body Therapies for Depression and Anxiety Symptoms in People with Cancer: A Systematic Review with Network Meta-Analysis
Source: Curr Oncol Rep. 2026 May 18;28(1):52. doi: 10.1007/s11912-026-01790-7 (PMC13183694; doi:10.1007/s11912-026-01790-7)
Supplement: Supplementary file 4 — Supplementary Material 4 (DOCX 59.5 KB) [file 11912_2026_1790_MOESM4_ESM.docx]

# Supplementary Material 4 - Studies Characteristics

Article title: Mind-body therapies for depression and anxiety symptoms in people with cancer: A systematic review with network meta-analysis

Journal name: Current Oncology Reports

Authors: Yoann Birling, Deep J. Bhuyan, Fan Feng, Jing Liu, Linda E. Carlson, Mingxian Jia, Wing Yu Yu, Han Zhang, Matthew Rahimi, Nibras Jasim, Betul H. Boge, Sarah Nevitt, Kayla Jaye, Indeewarie D. Mudiyanselage, Changrong Tang, Tiffany Tram, Judith Lacey, Rogier Hoenders, Paul P. Fahey.

Corresponding author: Yoann Birling, NICM Health Research Institute, Western Sydney University, [yoannbirling@gmail.com](mailto:yoannbirling@gmail.com).

This supplementary material presents the studies characteristics of the studies included in the systematic review, including population, interventions, and outcome measures.

| Article ID | Population | MBT intervention | Control intervention | Duration | Outcome measure |
| --- | --- | --- | --- | --- | --- |
| Alacacioglu 2018 | 98 Fm with breast cancer, mean age 51.7 years | Music listening, ind unsup F2F without HP | Usual care | NR | STAI |
| Arakawa 1995 | 24 Fm and 36 Ml with various cancers, mean age 57.1 years | Progressive muscular relaxation, three 25-min ind sup F2F sessions per day with HP | Usual care | 4 wks | STAI |
| Arruda 2016 | 47 Fm, 18 Ml and 10 Unk with various cancers | Music listening, one 30-min ind uns F2F daily session without HP | 1. Physical relaxation 2. Usual care | 3 days | BDI |
| Bahcaci 2021 | 44 Fm with breast cancer (stage 1, 2 and 3), mean age 59.7 years | Progressive muscular relaxation, one 60-min ind sup F2F session per week with HP | Usual care | 6 wks | HADS-A, HADS-D |
| Bahcaci 2024a | 64 Fm with breast cancer (mostly stage 3-4), mean age 46.9 years | Physical relaxation, three 40-min group sup rem session per week without HP | Waitlist | 6 wks | HADS-A, HADS-D |
| Bahcaci 2024b | 54 Fm with breast cancer (mostly stage 0-2), mean age 59.2 years | Physical relaxation, three 40-min group sup rem session per week without HP | Waitlist | 6 wks | HADS-A, HADS-D |
| Bahcivan 2022a | 173 Fm with breast cancer, mean age 52.3 years | Meditation, one single 20-min group sup F2F session without HP | CBT | 1 day | HADS-A HADS-D |
| Banerjee 2007 | 58 Fm with breast cancer (stage 2 and 3), mean age 44 years | Yoga (multicomponent), | Psychotherapy | 6 wks | HADS-A HADS-D |
| Baqer 2020 | 75 Fm and 84 Ml with various cancers, mean age 45.9 years | 1. Quran listening (spiritual intervention), one single 20-min ind unsup remote session without HP  2. Music listening, one single 20-min ind unsup remote session without HP | Usual care | 1 day | STAI |
| Blaes 2016 | 38 Fm and 4 Ml with various cancers, mean age 55 years | MBCR, one 150-min group sup F2F session per week with HP | Waitlist | 8 wks | STAI |
| Bower 2012 | 31 Fm with breast cancer (stage 0-2), mean age 54.9 years | Yoga (postures), one 90-min group sup F2F session per week without HP | Education | 12 wks | BDI |
| Bower 2015 | 71 Fm with breast cancer (stage 0-3), 50 years and younger | Meditation, one 120-min group sup F2F session per week with HP | Waitlist | 6 wks | CES-D |
| Bower 2021 | 247 Fm with breast cancer, mean age 45.4 years | Meditation, one 120-min group sup F2F session per week with HP | 1. Education  2. Usual care | 6 wks | CES-D |
| Branstrom 2010 | 70 Fm and 1 Ml with various cancers, mean age 51.8 years | MBSR, one 120-min group sup F2F session per week with HP | Waitlist | 8 wks | HADS-A, HADS-D |
| Bro 2019 | 63 Fm and 80 Ml with lymphoma, mean age 60 years | 1. Music listening, five 30-min ind unsup F2F sessions without HP  2. Music listening, thirty 5-min ind unsup remote sessions without HP | Usual care | 12 wks | STAI |
| Burch 2020 | 29 Fm, 5 Ml and 4 Unk with various cancers (stage 1-4), mean age 59.5 years | Biofeedback, one 25-min ind uns F2F session per week with HP | Usual care | 5 wks | BDI |
| Burns 2018 | 48 Fm and 28 Ml with various cancers, mean age 47.5 years | 1. Guided imagery with music, one single ind sup F2F session without HP  2. Music listening, one single 45-min ind unsup F2F session without HP | None | 1 day | HADS-A, HADS-D |
| Campbell-Gillies 2022 | 19 Fm and 11 Ml with various cancers (stage 1-3), mean age 60 years | Guided imagery, one ind unsup F2F session with HP | Usual care | 1 week | HAMA, HAMD |
| Cao DD 2020 | 52 Fm and 62 Ml with leukemia, mean age 33.1 years | MBSR, one 15-min ind sup F2F session per day without HP | Usual care | 8 wks | HADS-A, HADS-D |
| Carlson 2013 | 271 Fm with breast cancer (mostly stage 0-2), mean age 54.9 years | MBCR, one 90-min group sup F2F session per week without HP | 1. Support group  2. Education | 8 wks | POMS-A, POMS-D |
| Chan C 2007 | 30 Fm and 49 Ml with lung cancer (stage 3-4), mean age 62.9 years | Progressive muscular relaxation, one session every four weeks | Usual care | 4 wks | STAI |
| Chandwani 2010 | 61 Fm with breast cancer (mostly stage 0-2), mean age 49.5 years | Yoga (multicomponent), three 60-min group sup F2F sessions every two weeks with HP | Waitlist | 6 wks | STAI-S, CES-D |
| Chandwani 2014 | 178 Fm with breast cancer (mostly stage 0-2), mean age 51.9 years | Yoga (multicomponent), three 60-min ind sup F2F sessions per week with HP | 1. Sham yoga 2. Waitlist | 6 wks | CES-D, CES-D |
| Chang YC 2022 | 51 Fm with breast cancer (mostly stage 0-2), mean age 47.8 years | MBSR, one 120-min group sup F2F session per week with HP | Usual care | 6 wks | DASS-A, DASS-D |
| Charalambous 2016 | 104 Fm, 104 Ml and 28 Fm with various cancers (stage 3) | Integrative relaxation, four 27-min ind sup F2F sessions per week with HP | Usual care | 4 wks | BDI |
| Chen J 2019 | 60 Fm with breast cancer, mean age 45.3 years | MBSR, one 33-min ind sup F2F session per day | Usual care | 8 wks | HAMA, HAMD |
| Chen M 2017 | 36 Fm and 34 Ml with leukemia, mean age 53.3 years | Qigong with music listening, two 60-min ind sup F2F sessions per day with HP | Usual care | 4 wks | HAMA, HAMD |
| Chen SC 2019 | 100 Fm with breast cancer (mostly 0-2), mean 50.4 years | Music listening, one single 45-min ind unsup F2F session | Usual care | 1 day | HADS-A, HADS-D |
| Chen Z 2013 | 96 Fm with breast cancer (mostly stage 0-2), mean age 45.0 years | Qigong/Tai Chi, five 40-min group sup F2F sessions per week with HP | Waitlist | 38 days | CES-D |
| Cheng D 2021 | 45 Fm and 60 Ml with various cancers, mean age 65.6 years | Tai Chi, three 40-min group sup F2F session per week with HP | 1. Physical exercise  2. Physical exercise  3. Usual care | 12 wks | GAD-7, PHQ-9 |
| Cheng XZ 2019 | 52 Fm and 68 Ml with oesophageal cancer (mostly stage 3-4), age range 58-71 years | MBSR, 40-min ind sup F2F sessions with HP | Usual care | 8 wks | CES-D |
| Cheung 2003 | 19 Fm and 40 Ml with colorectal cancer, mean age 58.3 years | Progressive muscular relaxation, two 20-min ind sup F2F sessions per week with HP | Usual care | 1 week | STAI-S |
| Cheung 2021 | 14 Fm and 16 Ml with lung cancer (stage 3B and 4), mean age 60.5 years | Tai Chi, one 60-min group sup F2F session per week with HP | 1. Physical exercise  2. Activity tracking | 12 wks | HADS-A, HADS-D |
| Cheung 2022 | 17 Fm and 11 Ml with various cancers, mean age 69.3 years | Qigong/Tai Chi, one 90-min group sup F2F session per week with HP | Physical exercise | 16 wks | GDS |
| Chirico 2020 | 94 Fm with breast cancer (mostly 0-2), mean age 55.7 years | 1. Virtual reality, one single 20-min ind unsup F2F session without HP  2. Music listening, one single 20-min ind unsup F2F session without HP | None | 1 day | STAI-S, POMS-D |
| Clark 2006 | 24 Fm and 39 Ml with various cancers (mostly stage 0-2), mean age 57.8 years | Music listening, 45-min ind unsup F2F sessions without HP | Usual care | NR | HADS-A, HADS-D |
| Cohen 2020 | 40 Fm with breast cancer, mean age 57.3 years | Biofeedback, 20-min ind unsup remote sessions without HP | Physical exercise | NR | HADS-A, HADS-D |
| Colwell 2020 | 38 Fm and 7 Ml, mean age 59.6 years | 1. Participant-created chant, one single group sup F2F session without HP  2. Participant-selected chant, one single group sup F2F session without HP | Usual care | 1 day | STAI, POMS-D |
| Cramer 2015 | 40 Fm with breast cancer (mostly stage 0-2), mean age 49.2 years | Hatha Yoga (multicomponent), one 90-min group sup F2F session per week with HP | Waitlist | 12 wks | HADS-A, HADS-D |
| Cramer 2016 | 21 Fm and 33 Ml with colorectal cancer (mostly stage 0-2), mean age 68.3 years | Hatha Yoga (multicomponent), one 90-min group sup F2F session per week with HP | Waitlist | 10 wks | HADS-A, HADS-D |
| Culos-Reed 2006 | 36 Fm and 2 Ml with various cancers | Yoga (multicomponent), 75-min group sup F2F sessions without HP | Waitlist | 7 wks | POMS-A, POMS-D |
| Danhauer 2009 | 44 Fm with breast cancer (mostly stage 0-2), mean age 55.8 years | Yoga (multicomponent), one 75-min group sup F2F per week without HP | Waitlist | 10 wks | CES-D |
| Dhruva 2015 | 14 Fm and 2 Ml with various cancers, mean age 54.2 years | Yoga (pranayama), one 60-min mixed sup F2F session per week with HP | Waitlist | 4 wks | HADS-A, HADS-D |
| Duzgun 2020 | 12 Fm and 48 Fm with various cancers (stage 3-4) | Music listening, six 10-min ind sup F2F sessions without HP | Usual care | NR | STAI |
| Eaton 2021 | 31 Fm and 9 Ml with various cancers, mean age 52.7 years | Hypnotic recording, one 15-min ind unsup remote session per day without HP | Waitlist | 4 wks | PROMIS-A, PROMIS-D |
| Eaton 2022 | 89 Fm and 20 Ml with various cancers, mean age 55.7 years | 1. Hypnotic recording, one 16-min ind unsup remote session per day without HP  2. Visualisation/imagery, one 16-min ind unsup remote session per day without HP | None | 4 wks | PROMIS-A, PROMIS-D |
| Elkins 2008 | 51 Fm with breast cancer, mean age 57 years | Hypnotherapy, five 50-min ind sup F2F sessions per week with HP | Usual care | 5 wks | HADS-A, CES-D |
| Elyasi 2021 | 50 Fm with breast cancer, mean age 46 years | Hypnotherapy, one 60-min ind sup F2F session per week without HP | 1. CBT  2. Usual care | 8 wks | HADS-A, HADS-D |
| Eyigor 2018 | 42 Fm with breast cancer, mean age 51.9 years | Yoga (multicomponent), two 60-min group sup F2F sessions per week with HP | Usual care | 10 wks | BDI |
| Fink 2023 | 28 Fm and 14 Ml with various cancers (mostly stage 3-4), mean age 51.6 years | 1. Biofeedback (electro-encephalogram), eight 37.5-min ind unsup F2F sessions without HP  2. Mindfulness training, two 37.5-min group sup F2F sessions per week without HP | None | 5 wks | GAD-7, PHQ-8 |
| Foley 2010 | 89 Fm and 26 Ml with various cancers, mean age 55.2 years | MBCT, one 120-min group sup F2F session per week without HP | Waitlist | 8 wks | HAMA, HAMD |
| Franco 2020 | 36 Fm with breast cancer, mean age 41.3 years | Flow meditation, one 120-min group sup F2F session per week with HP | Waitlist | 7 wks | POMS-A |
| Gao R 2018 | 22 Fm and 38 Ml with lung cancer (mostly stage 3-4), mean age 53.9 years | MBSR, one 35-min ind sup F2F session per day without HP | Usual care | 6 wks | HAMA, HAMD |
| Gregoire 2020 | 95 Fm and 9 Ml with various cancers, mean age 53.9 years | Self-care and self-hypnosis, one 120-min group sup F2F session per week with HP | Waitlist | 8 wks | HADS-A, HADS-D |
| Gross 1995 | 30 Fm with breast cancer (stage 1-2), mean age 48.5 years | Guided imagery, two ind unsup F2F sessions per day without HP | Usual care | 8 wks | POMS-A |
| Gu 2024 | 103 Fm with breast cancer (mostly stage 0-2), mean age 46.8 years | MBCR, one 90-min group sup rem session per week with HP | Education | 4 wks | HADS-A, HADS-D |
| Hanser 2006 | 60 Fm with breast cancer (stage 4) | Music engagement, three 45-min group sup F2F sessions without HP | Usual care | NR | HADS-A, HADS-D |
| Harper 2001 | 33 Fm and 7 Ml with various cancers (mostly stage 3-4), mean age 52 years | 1. Problem-focused visualisation, one single 30-min ind sup F2F session without HP  2. Emotion-focused visualisation, one single 30-min ind sup F2F session without HP  3. Music listening, one single 30-min ind sup F2F session without HP | Usual care | 1 day | STAI-S |
| Hidderley 2004 | 31 Fm with breast cancer (stage 0-2), age range 16-65 years | Autogenic training (hypnotherapy), one group sup F2F session per day without HP | Usual care | 8 wks | HADS-A |
| Ho 2016 (bms) | 157 Fm with breast cancer (stage 1-3), mean age 47.8 years | Body-mind-spirit intervention, one 120-min group sup F2F session per week without HP | 1. supportive-expressive therapy  2. social support group | 8 wks | HADS-A, HADS-D |
| Ho 2016 (dance) | 139 Fm with breast cancer (mostly stage 0-2), mean age 48.8 years | Dance and movement therapy, two 90-min group sup F2F session per week without HP | Waitlist | 3 wks | HADS-A, HADS-D |
| Hoogland 2018 | 240 Fm with various cancers (mostly stage 0-2), mean age 51.4 years | Stress management program (multicomponent), four 10-min ind unsup remote sessions | Usual care | 13 wks | HADS-A, HADS-D |
| Huang YY 2007 | 48 Fm and 69 Ml with various cancers, mean age 30.3 years | Yoga (multicomponent), one 60-min ind sup F2F session every two days without HP | Usual care | 2 mts | STAI |
| Ioannou 2022 | 21 Fm and 29 Ml with cancer (mostly stage 3-4), mean age 57 years | 1. Virtual reality, one single 20-min ind sup F2F session without HP  2. Guided imagery, one single 20-min ind sup F2F session without HP | None | 1 day | POMS-A, POMS-D |
| Irwin 2017 | 90 Fm with breast cancer, mean age 59.8 years | Qigong/Tai Chi, one 120-min group sup F2F session per week without HP | CBT | 14 wks | IDS |
| Jain 2012 | 76 Fm with breast cancer (mostly stage 0-2), mean age 51.0 years | Reiki/touch therapy, two 52.5-min sup F2F sessions per week without HP | 1. Sham reiki/touch therapy 2. Waitlist | 4 wks | CES-D |
| Janusek 2019 | 164 Fm with breast cancer (mostly stage 0-2), mean age 55.1 years | MBSR, one 150-min group sup F2F session per week with HP plus 360-min retreat | Education | 8 wks | CES-D |
| Jaya 2020 | 50 individuals with cancer | Progressive muscular relaxation, three group sup F2F sessions per week without HP | Physical exercise | 3 wks | HADS-A |
| Jensens-Johansen 2013 | 437 Fm with breast cancer (stage 1-2), age range 27-70 years | Expressive writing (art therapy), one 20-min ind sup remote session per week with HP | Neutral writing | 3 wks | POMS-A, BDI |
| Johns 2016 | 64 Fm and 7 Ml with various cancers (mostly stage 0-2), mean age 56.6 years | MBSR, one 120-min group sup F2F session per week with HP | Psychotherapy | 8 wks | GAD-7, PHQ-8 |
| Jong 2018 | 83 Fm with breast cancer, mean age 51 years | Dru-based yoga (multicomponent), one 75-min group sup F2F session per week with HP | Waitlist | 12 wks | HADS-A, HADS-D |
| Juarez 2024 | 31 Fm with breast cancer, mean age 51.5 years | Other MBI, one 90-min group sup rem session per week without HP | Waitlist | 6 wks | GAD-7, CES-D |
| Kahveci 2025 | 31 Fm and 27 Ml with various cancers (mostly stage 3-4), mean age 49.3 years | 1. Reiki, one 27.5-min ind sup F2F session daily without HP  2. Physical relaxation, one 27.5-min ind sup F2F session daily without HP | Sham reiki | 4 days | STAI-S |
| Kiecolt-Glaser 2014 | 200 Fm with breast cancer (mostly stage 0-2), mean age 51.7 years | Yoga (multicomponent), two 90-min group sup F2F sessions per week with HP | Waitlist | 12 wks | CES-D |
| Kim 2005 | 18 Fm and 17 Ml with leukemia, mean age 36.6 years | Breathing relaxation, one 30-min group sup F2F session per week without HP | Usual care | 6 wks | STAI, BDI |
| Kim 2015 | 42 Fm and 24 Unk with various cancers | Laughter therapy, daily 60-min group sup F2F sessions without HP | Waitlist | 3 days | POMS-D |
| Kingston 2015 | 10 Fm and 6 Ml with various cancers, mean age 50 years | MBCT, one 90-min session per week without HP | Usual care | 8 wks | HADS-A, HADS-D |
| Knoerl 2022 | 42 Fm and 2 Ml with various cancers (mostly stage 3-4), mean age 60 years | Yoga (multicomponent), three 45-min mixed group sup F2F and ind unsup remote sessions every two weeks with HP | Usual care | 8 wks | POMS-A, POMS-D |
| Koca 2022 | 72 Fm and 20 Ml with various cancers (mostly stage 0-2) | Music listening, one single 60-min ind unsup F2F session without HP | Usual care | 1 day | STAI |
| Kremerer 2023 | 67 Ml with prostate cance, mean age 68.1 years | MBCT, one 120-min group sup F2F session per week without HP | 1. CBT  2. Usual care | 4 wks | HADS-A, HADS-D |
| Krischer 2007 | 222 Fm and 88 Ml with various cancers, mean age 60.9 years | Stress management program (multicomponent), ind unsup remote sessions without HP | Usual care | 3 wks | STAI-S, CES-D |
| Larkey 2015 | 101 Fm with breast cancer (mostly stage 0-2), mean age 58.8 years | Qigong/Tai Chi, one 60-min group sup F2F session per week with HP | Sham qigong/tai chi | 12 wks | BDI |
| Larkey 2022 | 101 Fm with breast cancer (mostly stage 0-2), mean age 58.8 years | Qigong/Tai Chi, one 60-min group sup F2F session per week with HP | Sham qigong | 12 wks | BDI |
| Lau BHP 2020 | 94 Fm and 63 Ml with lung cancer (mostly stage 3-4), mean age 60 years | Body-mind-spirit intervention, one 180-min group sup F2F session per week with home practice | CBT | 8 wks | HADS-A, HADS-D |
| Lee 2017 | 18 Fm with breast cancer, age range 35 to 67 years | MBSR, one 120-min group sup F2F session per week with HP | Waitlist | 8 wks | HADS-A, HADS-D |
| Lee 2020 | 36 Fm and 14 Unk with various cancers (mostly stage 0-2) | Laughter therapy, one 60-min group sup F2F session per week without HP | Usual care | 8 wks | BDI |
| Leite 2021 | 52 Fm and 23 Unk with breast cancer (stage 0-3), mean age 55.0 years | Dance/movements therapy, three 60-min group sup F2F sessions per week without HP | 1. Physical exercise 2. Usual care | 16 wks | BDI |
| Lengacher 2009 | 84 Fm with breast cancer (mostly stage 0-2), mean age 57.5 years | MBSR, one 120-min group sup F2F session per week with HP | Usual care | 6 wks | STAI-S, CES-D |
| Lengacher 2016 | 322 Fm with breast cancer (mostly stage 0-2), mean age 56.6 years | MBSR, one 120-min group sup F2F session per week with HP | Waitlist | 6 wks | STAI-S, CES-D |
| Lengacher 2025 | 212 Fm with breast cancer, mean age 56.5 years | MBSR, one 120-min group sup F2F session per week with HP | 1. Education  2. Usual care | 6 wks | PROMIS-A, PROMIS-D |
| Li WJ 2020 | 83 Fm with breast cancer (mostly stage 3-4), mean age 48.3 years | MBCR, one 90-min ind sup F2F session per week with HP | Usual care | 8 wks | HADS-A, HADS-D |
| Li XM 2012 | 120 Fm with breast cancer, mean age 45 years | Music listening, two 30-min in unsup F2F sessions per day without HP | Usual care | 8 wks | STAI-S |
| Lima 2020 | 33 Fm with breast cancer (stage 1-2), mean age 50.1 years | Music listening, one 30-min ind unsup F2F session per day without HP | Physical relaxation | 12 wks | BAI, BDI |
| Lin MF 2011a | 65 Fm and 23 Ml with various cancers, mean age 52.9 years | 1. Music listening, one 60-min ind unsup F2F session every two weeks without HP  2. Integrative relaxation, one 30-min ind unsup F2F session every two weeks without HP | Usual care | 4 wks | STAI-S |
| Lin MF 2011b | 65 Fm and 33 Ml with various cancers, mean age 52.9 years | 1. Integrative relaxation, one single 60-min group sup F2F session without HP  2. Integrative relaxation, one single 30-min ind unsup remote session without HP | Usual care | 1 day | STAI |
| Liossi 2001 | 23 Fm and 27 Ml with cancer, age range 35 to 74 years | Hypnotherapy, one 30-min ind sup F2F session per week without HP | Usual care | 4 wks | HADS-A |
| Liu CJ 2008 | 28 Fm and 21 Unk with breast cancer (stage 1-3), mean age 49.1 years | Body-mind-spirit intervention, one 180-min group sup F2F session per week without HP | Usual care | 10 wks | BDI |
| Liu J 2016 | 98 Fm with breast cancer (mostly stage 0-2), mean age 46.6 years | MBSR, one 120-min group sup F2F session per week with HP | Usual care | 4 wks | HADS-A, HADS-D |
| Liu Q 2022 | 106 Fm and 2 Ml with breast cancer (mostly stage 0-2), mean age 51.4 years | MBSR, one 45-min group sup remote session per week with HP | 1. Acupressure  2. Usual care | 8 wks | HADS-A, HADS-D |
| Liu W 2022 | 136 Fm with breast cancer (stage 1-2), age range 51 to 60 years | Mindfulness yoga (multicomponent), one 90-min group sup F2F session per week without HP | Usual care | 8 wks | HADS-A, HADS-D |
| Liu X 2020 | 34 Fm and 50 Ml with lung cancer, mean age 58.1 years | Music listening, 30-min ind uns F2F sessions without HP | Usual care | NR | HAMD |
| Liu Z 2022 | 28 Fm and 94 Ml with liver cancer, mean age 55.7 years | MBSR, five 20-min ind sup remote sessions per week with HP | Waitlist | 6 wks | HADS-A, HADS-D |
| Lopez 2023 | 33 Fm and 2 Ml with various cancers | Meditation app, one 10-min ind unsup remote session per day without HP | Waitlist | 2 wks | HADS-A, HADS-D |
| Lu N 2023 | 26 Fm and 38 Ml with lung cancer (stage 3-4), mean age 64.9 years | MBSR, one 37.5-min group sup F2F session every two days without HP | Usual care | 2 wks | HAMA, HAMD |
| Ma 2024 | 42 Fm with gynaecological cancers, mean age 43.7 years | Yoga, two 70-min group sup F2F sessions per week with HP | Usual care | 6 wks | HADS-A, HADS-D |
| Malik 2024 | 58 Fm with ovarian cancer (mostly stage 3-4), mean age 49.2 years | 1. Meditation, one daily 15-min ind unsuo rem session without HP  2. Music listening, one daily 15-min ind unsuo rem session without HP | None | 12 wks | HAMA |
| Mantoudi 2020 | 63 Fm and 17 Ml with various cancers, mean age 58.9 years | Integrative relaxation, one 30-min session per week without HP | Reflexology | 6 wks | HADS-A, HADS-D |
| Masoume 2021 | 49 Fm and 9 Ml with various cancers, age range 47 to 77 years | MBCT, one 90-min group sup F2F session per week without HP | 1. CBT  2. Waitlist | 8 wks | BAI, BDI |
| McCombie 2023 | 32 Fm and 36 Ml with digestive cancers (mostly stage 0-2), mean age 67.3 years | MBSR, one 120-min group sup F2F session per week without HP | Education | 4 wks | HADS-A, HADS-D |
| Mi Ra 2017 | 54 Fm with breast cancer (mostly stage 0-2), mean age 48.4 years | Mind subtraction meditation, two 120-min group sup F2F sessions per week without HP | Education | 8 wks | BAI, CES-D |
| Milbury 2013 | 47 Fm with breast cancer (mostly stage 0-2), mean age 53.5 years | Meditation, two 60-min group sup F2F sessions per week with HP | Waitlist | 6 wks | CES-D |
| Milbury 2019 | 20 Fm with glioma (mostly stage 3-4), mean age 46.3 years | Yoga (multicomponent), 12 45-min group sup F2F sessions without HP | Waitlist | 5 wks | CES-D |
| Molassiotis 2002 | 92 Fm with breast cancer (mostly stage 0-2), mean age 45 years | Progressive muscular relaxation, one single 30-min ind sup F2F with HP | Usual care | 2 wks | STAI, POMS-D |
| Molassiotis 2021 | 40 Fm and 116 Ml with lung cancer (mostly stage 3-4), mean age 56.8 years | Qigong, four 60-min group sup F2F sessions per week with HP | Waitlist | 6 wks | DASS-A |
| Monti 2013 | 191 Fm and 61 Unk with breast cancer (mostly stage 0-2), mean age 53.6 years | Art therapy, one 150-min group sup F2F session per week with HP | Education | 8 wks | SCL-90-D |
| Mosher 2024 | 34 Fm and 21 Ml with various cancers (only stage 3-4), mean age 70.9 years | MBSR, one 120-min group sup F2F session per week with HP | Usual care | 6 wks | GAD-7, PHQ-8 |
| Nakamura 2013 | 43 Fm and 14 Ml with various cancers, mean age 52.6 years | 1. Mind-body bridging (meditation), one 120-min group sup F2F session per week with HP 2. MBSR, one 120-min group sup F2F session per week with HP | Education | 3 wks | CES-D |
| Napoles 2020 | 153 Fm with breast cancer (mostly stage 0-2), mean age 54.8 years | Multicomponent MBT, one 90-min ind sup F2F session per week with home practice | Waitlist | 10 wks | PHQ-8 |
| Ngamkham 2017 | 15 Fm and 2 Ml with various cancers (mostly stage 0-2), mean age 50 years | Thai Buddhism-based meditation, three group sup F2F sessions with HP | Usual care | 8 wks | HADS-A, HADS-D |
| Nguyen 2023 | 21 Fm with various cancers (mostly stage 3-4), mean age 52.3 years | Integrative relaxation, one daily 40-min ind unsup rem session without HP | Usual care | 3 wks | DASS-A, DASS-D |
| Nissen 2019 | 137 Fm and 13 Ml with various cancers, mean age 55.7 years | MBCT, eight ind unsup remote sessions without HP | Waitlist | 10 wks | STAI, BDI |
| Noh Gie 2011 | 63 Fm with gynaecological cancers, mean age 53.2 years | Music engagement, one single group sup F2F session without HP | Usual care | 1 day | STAI-S |
| Nuzhath 2024 | 70 Fm with cervical cancer, mean age 52.3 years | Yoga, ten 30-min group sup F2F sessions per week without HP | Usual care | 6 wks | HADS-A, HADS-D |
| O'Callaghan 2012 | 41 Fm and 59 Ml with various cancers, mean age 57.5 years | Music listening, ind unsup F2F sessions without HP | Usual care | NR | STAI-S |
| Pan YN 2017 | 41 Fm and 54 Ml with gastrointestinal cancers | Music listening, three 25-min ind uns F2F sessions every two weeks without HP | Usual care | 8 wks | HAMD |
| Park 2020 | 76 Fm with breast cancer (mostly stage 0-2), mean age 53.7 years | MBCT, one 120-min group sup F2F session per week with HP | Waitlist | 8 wks | HADS-A, HADS-D |
| Potthoff 2012 | 160 Fm with breast cancer (mostly stage 0-2), mean age 55.8 years | Physical relaxation, two 60-min sessions per week without HP | Physical exercise | 12 wks | CES-D |
| Pouy 2018 | 70 Fm with breast cancer, mean age 54.1 years | MBSR, two 90-min group sup F2F sessions per week without HP | Waitlist | 4 wks | DASS-A, DASS-D |
| Puig 2006 | 33 Fm with breast cancer (stage 1-2), mean age 51.4 years | Art therapy, one 60-min group sup F2F session per week without HP | Waitlist | 4 wks | POMS-A, POMS-D |
| Qiao Li 2018 | 32 Fm and 56 Ml with various cancers (mostly stage 3-4), mean age 56.8 years | Breathing and muscular relaxation, imagery and music listening, two 45-min ind sup F2F sessions per week without HP | Usual care | 4 wks | HAMA, HAMD |
| Qin J 2016 | 17 Fm and 33 Ml with leukemia, mean age 37.4 years | Linden relaxation technique, one 30-min ind sup F2F session per day without HP | Usual care | 2 wks | HAMA |
| Rabinowitch 2023 | 28 Fm and 2 Ml with various cancers, mean age 54.3 years | 1. Music, one single 60-min group sup rem session without HP  2. Meditation, one single 60-min group sup rem session without HP | None | 1 day | STAI-6 |
| Raghavendra 2009 | 88 Fm with breast cancer (mostly stage 3-4), mean age 47.2 years | Yoga (multicomponent), three 60-min group sup F2F sessions per week with HP | Psychotherapy | 6 wks | HADS-A, HADS-D |
| Rao 2009 | 98 Fm with breast cancer (stage 2-3) | Yoga (multicomponent), ind sup F2F sessions with HP | Psychotherapy | NR | STAI-S |
| Sabo 1996 | 58 Fm and 39 Ml with various cancers | Music listening, ind unsup F2F sessions without HP | Usual care | 16 wks | STAI-S |
| Sarenmalm 2017 | 177 Fm with breast cancer, mean age 57.2 years | 1. MBSR, one 120-min group sup F2F session per week with HP 2. MBSR, ind uns rem sessions | Usual care | 8 wks | HADS-D |
| Schroder 2022 | 51 Fm with breast cancer, mean age 55.8 years | Mindful walking, one 90-min group sup F2F session per week with HP | Physical exercise | 8 wks | HADS-A, HADS-D |
| Shan MS 2019 | 90 Fm with breast cancer (mostly stage 0-2), mean age 45.4 years | MBSR, one 45-min group sup F2F session per day with HP | Usual care | 6 wks | GAD-7, PHQ-9 |
| Shao D 2020 | 144 Fm with breast cancer (mostly stage 0-2), mean age 42.3 years | MBSR, one und sup remote session per week with HP | Waitlist | 6 wks | GAD-7 |
| Sharpe 2019 | 145 Fm and 7 Ml with various cancers (stage 0-3), mean age 52.8 years | Integrative relaxation, one 75-min ind sup F2F session every two weeks with HP | Psychotherapy | 10 wks | DASS-A, DASS-D |
| Shelley 2014 | 33 Fm and 2 Ml with various cancers (mostly stage 0-2), mean age 57.3 years | MBSR, one 120-min group sup F2F session per week with HP | Waitlist | 8 wks | GAD-7, PHQ-8 |
| Shergill 2022 | 98 Fm with breast cancer, mean age 52.7 years | MBSR, one 150-min group sup F2F session per week without HP | Waitlist | 8 wks | POMS-A, PHQ-9 |
| Smith 2001 | 42 Ml with prostate cancer, mean age 62.8 years | Music listening, 20-min ind unsup F2F sessions without HP | Usual care | 5 wks | STAI |
| Sohl 2022 | 21 Fm and 23 Ml with gastrointestinal cancer (mostly stage 3-4), mean age 58.5 years | Yoga (multicomponent), one 30-min sup F2F session every two weeks with HP | Attention control | 8 wks | PROMIS-D |
| Song QH 2013 | 100 Fm with breast cancer, mean age 43.6 years | Physical relaxation, ind sup F2F sessions without HP | Usual care | NR | STAI |
| Speca 2000 | 73 Fm and 17 Ml with various cancers, mean age 51 years | MBSR, one 90-min group sup F2F per week with HP | Waitlist | 7 wks | POMS-A, POMS-D |
| Sun J 2018 | 37 Fm and 45 Ml with leukemia, mean age 44.2 years | Emotional care and relaxation, 15-min ind sup F2F sessions without HP | Usual care | NR | HAMA, HAMD |
| Targ 2002 | 157 Fm with breast cancer, age range 26 to 78 years | Multicomponent MBT, two 150-min group sup F2F sessions per week without HP | Support group | 12 wks | POMS-A, POMS-D |
| Taylor 2018 | 33 Fm with breast cancer, mean age 53.8 years | Yoga (multicomponent), one 75-min group sup F2F session per week without HP | Waitlist | 8 wks | CES-D |
| Thyme 2009 | 41 Fm and 1 Unk with breast cancer, age range 37 to 69 years | Art therapy, one group sup F2F session per week without HP | Usual care | 5 wks | SCL-90-D |
| Vargas-Roman 2022 | 19 Fm and 20 Ml with lymphoma (mostly stage 0-2), mean age 44.5 years | Qigong, two 60-min group sup F2F sessions per week with HP | Education | 8 wks | HADS-A, HADS-D |
| Vaziri 2017 | 20 Fm with breast cancer | MBCT, one 120-min session per week without HP | Waitlist | 8 wks | DASS-A, DASS-D |
| Victorson 2020 | 99 Fm and 27 Ml with various cancers, mean age 32.8 years | MBSR, one 150-min group sup F2F session per week without HP | Waitlist | 8 wks | PROMIS-A, PROMIS-D |
| Victorson 2024 | 46 Ml with prostate cancer (mostly stage 0-2), mean age 64.1 years | 1. Meditation, one daily 7-min ind unsup F2F session without HP  2. Music listening, one daily 7-min ind unsup F2F session without HP | None | 1 week | PROMIS-A, PROMIS-D |
| Walker 1997 | 96 Fm with breast cancer (mostly stage 0-2), mean age 49.7 years | Progressive muscular relaxation and guided imagery, one group sup F2F session per day with HP | Usual care | 18 wks | HADS-A |
| Wan YH 2009 | 76 Fm and 60 Ml with various cancers, mean age 52.5 years | Music listening, three 30-min ind unsup F2F sessions per week without HP | Usual care | 4 wks | STAI-S, CES-D |
| Wen 2022 | 21 Fm and 67 Ml with head and neck cancer (mostly stage 3-4), mean age 46.3 years | Baduanjin qigong, one 40-min ind unsup remote session per week without HP | Usual care | 12 wks | GAD-7, PHQ-9 |
| Wen MH 2016 | 70 Fm with gynaecological cancers, mean ag 51.2 years | Traditional music listening, six 25-min ind unsup F2F sessions without HP | Usual care | NR | HAMA |
| Wong 2024 | 35 Fm with breast cancer (mostly stage 0-2), mean age 47.2 years | Yoga, one 60-min group sup F2F session per week without HP | Waitlist | 8 wks | HADS-A, HADS-D |
| Wu GX 2020 | 39 Fm and 75 Ml with lung cancer (mostly stage 0-2), mean age 67.5 years | MBSR, one 30-min sup F2F session per day with HP | Usual care | 8 wks | HAMA, HAMD |
| Wu Xiuying 2015 | 138 individuals with liver cancer, age range 19-65 years | Music listening, 30-min ind unsup F2F sessions without HP | Usual care | NR | STAI-S |
| Wurtzen 2013 | 336 Fm with breast cancer (mostly stage 0-2), mean age 54.1 years | MBSR, one 120-min group sup F2F session per week with HP plus retreat | Usual care | 8 wks | CES-D |
| Xu HY 2018 | 37 Fm, 79 Ml and 4 Unk with liver cancer, mean age 61.5 years | Music listening, two 30-min ind uns F2F sessions per day without HP | 1. Chinese herbal medicine 2. Usual care | 45 days | HAMD |
| Xu YL 2018 | 84 Fm with breast cancer, mean age 44.1 years | Music listening, two 60-min ind unsup F2F sessions per day without HP | Usual care | 1 week | HAMA |
| Yang 2010 | 29 Fm and 61 Ml with nasopharyngeal cancer (mostly stage 3) | 1. Muscular relaxation and imagery, one 30-min ind sup F2F session per day without HP  2. Calligraphy (art therapy) , one 30-min ind sup F2F session per day without HP | Usual care | 4 wks | POMS-A, POMS-D |
| Yao 2022 | 72 Fm with breast cancer (mostly stage 0-2), mean age 46.9 years | Tai Chi, two 60-min group sup F2F sessions per week with HP | Usual care | 8 wks | HADS-D |
| Yildrim 2024 | 36 Fm with various cancers, mean age 58.1 years | Multicomponent MBT, one 45-min group sup F2F session every three days without HP | Attention control | 9 days | STAI |
| Ying W 2019 | 100 Fm with breast cancer | Baduanjin qigong, three 60-min group sup F2F sessions per day with HP | Usual care | 4 wks | STAI, PHQ-9 |
| You M 2020 | 148 Fm and 230 Ml with lung cancer (stage 0-2), mean age 58.1 years | MBSR, one 120-min group sup F2F session per day without HP | Usual care | 6 wks | HADS-A, HADS-D |
| Yun X 2014 | 36 Fm with breast cancer (mostly stage 0-2) | Progressive muscular relaxation, 12.5-min ind sup F2F sessions without HP | Usual care | NR | HAMA |
| Zhang 2023 | 33 Fm and 27 Ml with leukemia, mean age 34.4 years | Meditation, one 20-min daily ind unsup F2F session without HP | Usual care | 2 wks | STAI-S, CES-D |
| Zhang JY 2017 | 58 Fm with breast cancer (stage 1-3), mean age 46.1 years | MBSR, one 120-min group sup F2F session per week with HP | Usual care | 8 wks | STAI |
| Zhang R 2020 | 128 Fm with cervical cancer (stage 0-2), mean age 41.7 years | Mindfulness meditation, one 45-min ind sup F2F sessions per week without HP | Usual care | 6 mts | BAI |
| Zhang XA 2011 | 48 Fm with breast cancer, mean age 48.3 years | Physical relaxation, one ind sup F2F session every two days without HP | Usual care | 12 wks | HAMA, HAMD |
| Zhao PT 2008 | 21 Fm and 28 Ml with various cancers, mean age 54.1 years | Music listening, one single 30-min ind unsup F2F session without HP | Usual care | 1 day | HAMA |
| Zhi 2021 | 41 Fm with various cancers, mean age 61.7 years | Yoga (multicomponent), one 60-min mixed sup mixed session per day with HP | Waitlist | 8 wks | HADS-A, HADS-D |
| Zhou KN 2011 | 120 Fm with breast cancer, age range 25 to 65 years | Music listening, 30-min ind unsup F2F sessions without HP | Usual care | NR | STAI-S |
| Zhu WH 2012 | 32 Fm and 58 Ml with various cancers, mean age 50.6 years | Music listening, ind uns F2F sessions without HP | Usual care | 4 wks | HAMD |
| Zhuang XQ 2020 | 94 Ml with prostate cancer, mean age 67.3 years | MBSR, daily 25-min ind sup F2F sessions with HP | Usual care | 8 wks | SCL-90-D |
| Zhuo W 2017 | 48 Fm and 58 Ml with gastrointestinal cancer, mean age 58.3 years | Music listening, daily 30-min ind uns F2F sessions without HP | Usual care | 4 wks | HAMD |

BAI = Beck Anxiety Index, BDI = Beck Depression Index, CES-D = Center for Epidemiological Studies Depression scale, DASS-A = Depression Anxiety and Stress Scale – Anxiety subscale, DASS-D = Depression Anxiety and Stress Scale – Depression subscale, F2F = face-to-face, GAD-7 = Generalised Anxiety Disorder – 7 items, GDS = Geriatric Depression Scale, HADS-A = Hospital Anxiety and Depression Scale – Anxiety subscale, HADS-D = Hospital Anxiety and Depression Scale – Depression subscale, HAMA = Hamilton Anxiety Rating Scale, HAMD = Hamilton Depression Rating Scale, HP = home practice, ind = individual, Ml = males, mts = months, Fm = females, MBCR = Mindfulness-Based Cancer Recovery, MBCT = Mindfulness-Based Cognitive Therapy, MBSR = Mindfulness-Based Stress Reduction, NR = not reported, PHQ = Patient Health Questionnaire, POMS-A = Profile of Mood States – Anxiety subscale, POMS-D = Profile of Mood States – Depression subscale, PROMIS-A = Patient-Reported Outcomes Measurement Information System – Anxiety subscale, PROMIS-D = Patient-Reported Outcomes Measurement Information System – Depression subscale, SCL-90-D = Symptom Checklist-90 Depression subscale, STAI = State-Trait Anxiety Scale, STAI-S = State-Trait Anxiety Scale – State subscale, sup = supervised, unsup = unsupervised, wks = weeks.
