# Supplementary material for: Mind-Body Therapies for Depression and Anxiety Symptoms in People with Cancer: A Systematic Review with Network Meta-Analysis
Source: Curr Oncol Rep. 2026 May 18;28(1):52. doi: 10.1007/s11912-026-01790-7 (PMC13183694; doi:10.1007/s11912-026-01790-7)
Supplement: Supplementary file 5 — Supplementary Material 5 (DOCX 216 KB) [file 11912_2026_1790_MOESM5_ESM.docx]

# Supplementary Material 6 – Network meta-analysis of acceptability

Article title: Mind-body therapies for depression and anxiety symptoms in people with cancer: A systematic review with network meta-analysis

Journal name: Current Oncology Reports

Authors: Yoann Birling, Deep J. Bhuyan, Fan Feng, Jing Liu, Linda E. Carlson, Mingxian Jia, Wing Yu Yu, Han Zhang, Matthew Rahimi, Nibras Jasim, Betul H. Boge, Sarah Nevitt, Kayla Jaye, Indeewarie D. Mudiyanselage, Changrong Tang, Tiffany Tram, Judith Lacey, Rogier Hoenders, Paul P. Fahey.

Corresponding author: Yoann Birling, NICM Health Research Institute, Western Sydney University, [yoannbirling@gmail.com](mailto:yoannbirling@gmail.com).

This supplementary presents the results of the network meta-analysis of acceptability, represented by the completion rate in the study. The results of the local and global inconsistency assessments are presented at the end of this supplementary material.

The network metaanalysis was conducted on risk ratio (RR) of proportion of people in the first treatment group who completed study divided by the proportion of people in the second treatment group who completed the study. The results of the network meta-analysis are listed below. RR’s above 1.0 suggest that these treatments are more acceptable (less dropouts) than usual care (i.e. lower drop out in this treatment group than in the usual care group). RR below 1.0 suggest these treatments are less acceptable than usual care (i.e. higher drop out in this treatment group than in the usual care group).

The completion rate of 234 pairwise comparisons from 184 studies was available and included in the network meta-analysis. The only treatment options displaying statistically significant differences in acceptability from usual care are ‘wt=wait list’ which is more acceptable than usual care (people don’t drop out while waiting for treatment) and ‘psyc=psychotherapy’ and ‘MBs= MBCR/MBCT/MBSR’ which have statistically significantly lower completion rates than usual care (the larger treatment groups having higher statistical power to detect differences from usual care).

Number of studies: k = 184

Number of pairwise comparisons: m = 234

Number of treatments: n = 34

Number of designs: d = 75

Random effects model

Treatment estimate (sm = 'RR', comparison: other treatments vs 'UC'):

RR 95%-CI z p-value

acp 1.0220 [0.9469; 1.1032] 0.56 0.5760

act 0.9516 [0.8330; 1.0872] -0.73 0.4657

art 1.0276 [0.9433; 1.1193] 0.62 0.5334

att 0.9517 [0.8172; 1.1084] -0.64 0.5247

bio 1.0143 [0.9142; 1.1254] 0.27 0.7886

bms 0.9720 [0.8814; 1.0719] -0.57 0.5692

CBT 1.0256 [0.9683; 1.0863] 0.86 0.3887

CHM 0.9387 [0.8440; 1.0439] -1.17 0.2431

danc 1.0594 [0.9477; 1.1842] 1.01 0.3104

ed 0.9712 [0.9305; 1.0136] -1.34 0.1799

ex 1.0177 [0.9506; 1.0895] 0.50 0.6141

hyp 1.0386 [0.9648; 1.1180] 1.01 0.3138

img 0.9762 [0.9187; 1.0374] -0.78 0.4381

irx 0.9702 [0.9255; 1.0171] -1.26 0.2089

joh 0.5797 [0.2644; 1.2708] -1.36 0.1733

lau 0.8493 [0.7156; 1.0080] -1.87 0.0617

MBs 0.9767 [0.9607; 0.9928] -2.82 0.0049

mMBT 0.9731 [0.9384; 1.0091] -1.47 0.1408

mus 1.0039 [0.9840; 1.0242] 0.38 0.7024

mwk 0.9367 [0.6901; 1.2716] -0.42 0.6751

prx 1.0104 [0.9626; 1.0607] 0.42 0.6747

psyc 0.9084 [0.8480; 0.9732] -2.74 0.0062

ref 0.9939 [0.8900; 1.1100] -0.11 0.9134

rek 0.9849 [0.9043; 1.0727] -0.35 0.7274

rtw 1.0003 [0.9318; 1.0737] 0.01 0.9944

spt 0.9736 [0.8570; 1.1060] -0.41 0.6809

sqTC 1.0199 [0.9032; 1.1516] 0.32 0.7509

srk 0.9846 [0.8408; 1.1530] -0.19 0.8474

sup 0.9351 [0.8701; 1.0051] -1.82 0.0685

syg 1.0445 [0.9368; 1.1645] 0.78 0.4334

TC 1.0102 [0.9795; 1.0417] 0.64 0.5201

UC . . . .

wt 1.0278 [1.0017; 1.0545] 2.09 0.0366

yog 1.0196 [0.9848; 1.0557] 1.10 0.2729

Quantifying heterogeneity / inconsistency:

tau^2 = 0.0008; tau = 0.0282; I^2 = 31% [16.7%; 42.9%]

Tests of heterogeneity (within designs) and inconsistency

(between designs):

Q d.f. p-value

Total 255.18 176 < 0.0001

Within designs 177.95 111 < 0.0001

Between designs 77.24 65 0.1423


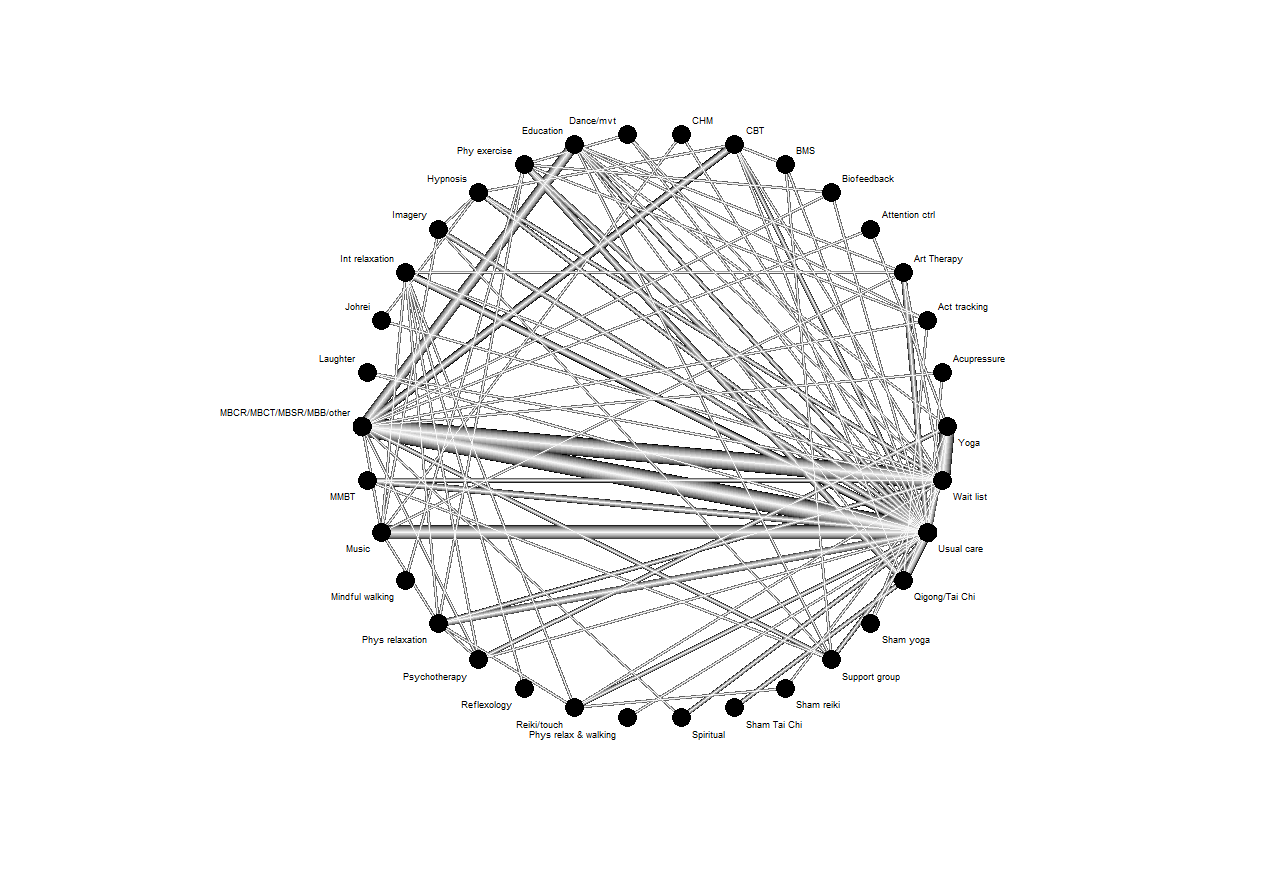


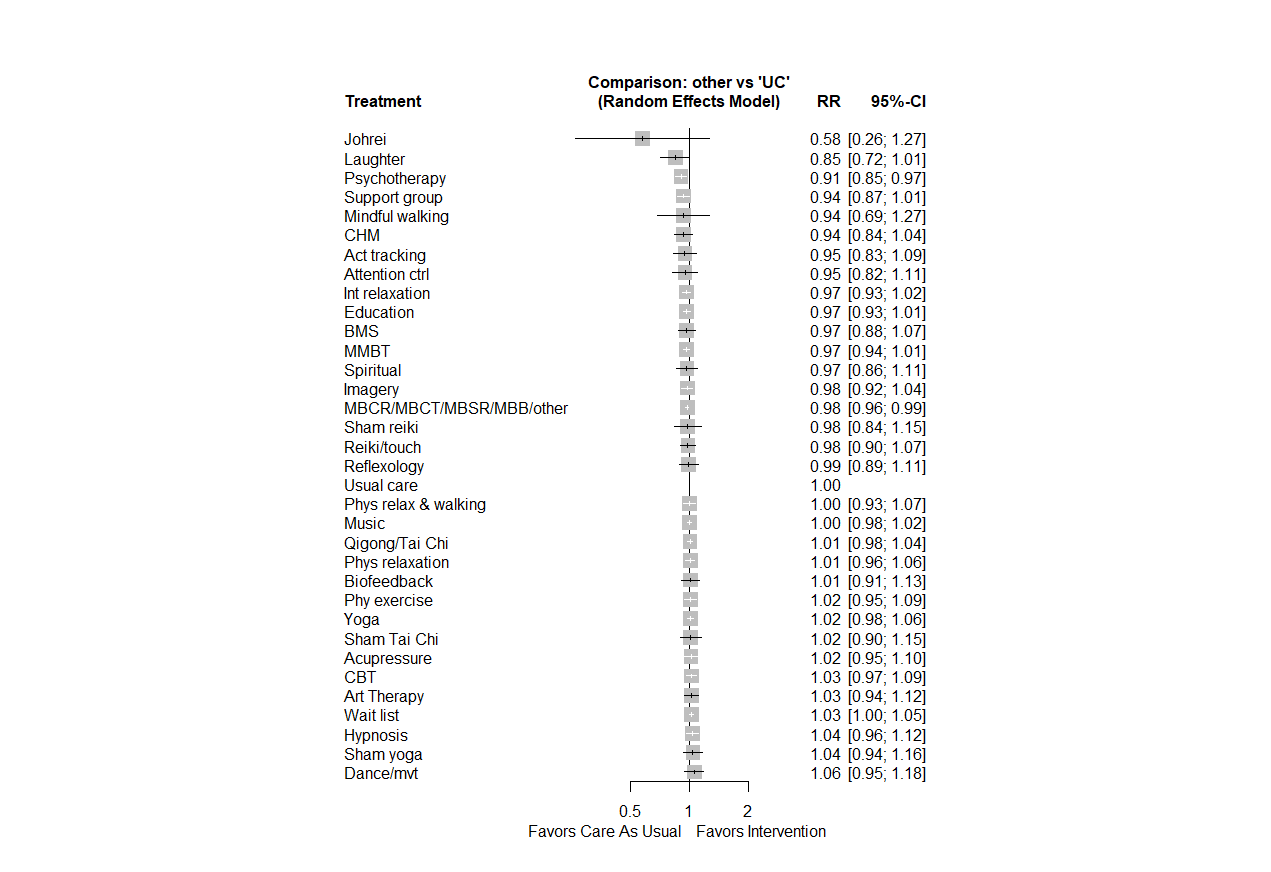


SUCRA and P-score ranks for each treatment.

|  | SUCRA |  | P-score |
| --- | --- | --- | --- |
| danc | 0.8135 | danc | 0.8156 |
| hyp | 0.7790 | hyp | 0.7776 |
| syg | 0.7683 | wt | 0.7696 |
| wt | 0.7679 | syg | 0.7639 |
| CBT | 0.7239 | CBT | 0.7270 |
| yog | 0.7051 | art | 0.7110 |
| acp | 0.7022 | yog | 0.7075 |
| art | 0.6872 | acp | 0.6893 |
| ex | 0.6642 | ex | 0.6723 |
| sqTC | 0.6630 | sqTC | 0.6489 |
| TC | 0.6395 | TC | 0.6415 |
| prx | 0.6360 | prx | 0.6364 |
| bio | 0.6333 | bio | 0.6320 |
| mus | 0.5878 | mus | 0.5958 |
| rtw | 0.5755 | rtw | 0.5633 |
| UC | 0.5614 | UC | 0.5603 |
| ref | 0.5312 | ref | 0.5284 |
| srk | 0.4922 | srk | 0.4909 |
| rek | 0.4596 | rek | 0.4727 |
| spt | 0.4388 | spt | 0.4344 |
| img | 0.4118 | bms | 0.4118 |
| bms | 0.4099 | img | 0.4070 |
| mwk | 0.4010 | mwk | 0.3867 |
| MBs | 0.3750 | MBs | 0.3792 |
| att | 0.3661 | mMBT | 0.3676 |
| mMBT | 0.3617 | ed | 0.3594 |
| ed | 0.3574 | irx | 0.3576 |
| irx | 0.3519 | att | 0.3567 |
| act | 0.3459 | act | 0.3415 |
| CHM | 0.2646 | CHM | 0.2639 |
| sup | 0.2084 | sup | 0.2092 |
| psyc | 0.1285 | psyc | 0.1294 |
| lau | 0.0994 | lau | 0.0962 |
| joh | 0.0887 | joh | 0.0952 |

On the above table ' Dance/movements therapy' has the highest retention rates relative to the associated controls, followed by hypnosis, sham yoga and waitlist. Laughter therapy and Johrei have the lowest retention relative to their associated controls.
