# Supplementary material for: Mind-Body Therapies for Depression and Anxiety Symptoms in People with Cancer: A Systematic Review with Network Meta-Analysis
Source: Curr Oncol Rep. 2026 May 18;28(1):52. doi: 10.1007/s11912-026-01790-7 (PMC13183694; doi:10.1007/s11912-026-01790-7)
Supplement: Supplementary file 6 — Supplementary Material 6 (DOCX 78.0 KB) [file 11912_2026_1790_MOESM6_ESM.docx]

# Supplementary Material 5 - Risk of bias of the included studies

Article title: Mind-body therapies for depression and anxiety symptoms in people with cancer: A systematic review with network meta-analysis

Journal name: Current Oncology Reports

Authors: Yoann Birling, Deep J. Bhuyan, Fan Feng, Jing Liu, Linda E. Carlson, Mingxian Jia, Wing Yu Yu, Han Zhang, Matthew Rahimi, Nibras Jasim, Betul H. Boge, Sarah Nevitt, Kayla Jaye, Indeewarie D. Mudiyanselage, Changrong Tang, Tiffany Tram, Judith Lacey, Rogier Hoenders, Paul P. Fahey.

Corresponding author: Yoann Birling, NICM Health Research Institute, Western Sydney University, [yoannbirling@gmail.com](mailto:yoannbirling@gmail.com).

This supplementary material presents the risk of bias of the studies included in the systematic review according to the type of control intervention (i.e., active or inactive).

A total of 182 studies were included in the meta-analysis. In the 61 studies in which MBT was compared to an active control (such as exercise or education), the overall risk of bias was ‘low’ in 9 studies, ‘some concerns’ in 44 studies, and ‘high’ in 8 studies. Concerns over bias were mainly concentrated in the measurement of the outcome, for which only 15 studies had a low risk of bias. All the 139 studies in which MBT was compared to an inactive control (such as usual care or waitlist), had an overall risk of bias that was high. This stems from the fact that blinding between an active intervention such as MBT and an inactive control such as usual care or waitlist is impossible.

| Study ID | Active control | | | | | | Inactive control | | | | | | |
| --- | --- | --- | --- | --- | --- | --- | --- | --- | --- | --- | --- | --- | --- |
|  | Randomi-sation bias | Interven-tions bias | Missing outcome bias | Measure-ment bias | Result selection bias | Overall bias | Randomi-sation bias | Interven-tions bias | Missing outcome | Measure-ment bias | Result selection | Overall bias |  |
| Alacacioglu 2018 | N/A | N/A | N/A | N/A | N/A | N/A | SC | Low | SC | High | SC | High |  |
| Arakawa 1995 | N/A | N/A | N/A | N/A | N/A | N/A | SC | Low | High | High | Low | High |  |
| Arruda 2016 | SC | Low | SC | SC | Low | SC | SC | Low | SC | High | Low | High |  |
| Bahcaci 2021 | Low | Low | Low | High | SC | High | N/A | N/A | N/A | N/A | N/A | N/A |  |
| Bahçaci 2024a | N/A | N/A | N/A | N/A | N/A | N/A | Low | Low | SC | High | Low | High |  |
| Bahçacı 2024b | N/A | N/A | N/A | N/A | N/A | N/A | SC | SC | SC | High | Low | High |  |
| Bahcivan 2022a | SC | low | low | SC | low | SC | N/A | N/A | N/A | N/A | N/A | N/A |  |
| Banerjee 2007 | SC | Low | SC | SC | Low | SC | N/A | N/A | N/A | N/A | N/A | N/A |  |
| Baqer 2020 | Low | Low | Low | Low | Low | Low | N/A | N/A | N/A | N/A | N/A | N/A |  |
| Blaes 2016 | N/A | N/A | N/A | N/A | N/A | N/A | SC | Low | SC | High | Low | High |  |
| Bower 2012 | Low | Low | Low | SC | Low | SC | N/A | N/A | N/A | N/A | N/A | N/A |  |
| Bower 2015 | N/A | N/A | N/A | N/A | N/A | N/A | Low | Low | Low | High | Low | High |  |
| Bower 2021 | low | low | low | SC | low | SC | low | low | low | high | low | high |  |
| Branstrom 2010 | N/A | N/A | N/A | N/A | N/A | N/A | High | Low | Low | High | Low | High |  |
| Bro 2019 | Low | SC | Low | SC | Low | SC | Low | SC | Low | High | Low | High |  |
| Burch 2020 | N/A | N/A | N/A | N/A | N/A | N/A | Low | Low | Low | High | Low | High |  |
| Burns 2018 | SC | Low | Low | SC | SC | SC | N/A | N/A | N/A | N/A | N/A | N/A |  |
| Campbell-Gillies 2022 | N/A | N/A | N/A | N/A | N/A | N/A | Low | Low | Low | High | SC | High |  |
| Cao DD 2020 | N/A | N/A | N/A | N/A | N/A | N/A | Low | Low | SC | High | SC | High |  |
| Carlson 2013 | Low | Low | Low | Low | Low | Low | N/A | N/A | N/A | N/A | N/A | N/A |  |
| Chandwani 2010 | N/A | N/A | N/A | N/A | N/A | N/A | Low | Low | Low | High | SC | High |  |
| Chandwani 2014 | Low | Low | Low | SC | Low | SC | Low | Low | Low | High | Low | High |  |
| Chang YC 2022 | N/A | N/A | N/A | N/A | N/A | N/A | SC | Low | Low | High | SC | High |  |
| Charalambous 2016 | N/A | N/A | N/A | N/A | N/A | N/A | Low | Low | Low | High | Low | High |  |
| Chen J 2019 | N/A | N/A | N/A | N/A | N/A | N/A | Low | Low | SC | High | SC | High |  |
| Chen M 2017 | N/A | N/A | N/A | N/A | N/A | N/A | Low | Low | SC | High | SC | High |  |
| Chen SC 2019 | N/A | N/A | N/A | N/A | N/A | N/A | SC | Low | Low | High | SC | High |  |
| Chen Z 2013 | N/A | N/A | N/A | N/A | N/A | N/A | Low | Low | Low | High | Low | High |  |
| Cheng D 2021 | Low | Low | Low | SC | Low | SC | Low | Low | Low | High | Low | High |  |
| Cheng XZ 2019 | N/A | N/A | N/A | N/A | N/A | N/A | Low | Low | SC | High | SC | High |  |
| Cheung 2003 | N/A | N/A | N/A | N/A | N/A | N/A | SC | Low | Low | High | SC | High |  |
| Cheung 2021 | Low | Low | Low | SC | Low | SC | Low | Low | Low | High | Low | High |  |
| Cheung 2022 | Low | Low | SC | SC | Low | SC | N/A | N/A | N/A | N/A | N/A | N/A |  |
| Chirico 2020 | SC | Low | Low | SC | SC | SC | SC | Low | Low | High | SC | High |  |
| Clark 2006 | N/A | N/A | N/A | N/A | N/A | N/A | Low | Low | Low | High | Low | High |  |
| Cohen 2004 | N/A | N/A | N/A | N/A | N/A | N/A | Low | Low | Low | High | SC | High |  |
| Cole 2012 | SC | Low | SC | Low | SC | SC | SC | Low | SC | High | SC | High |  |
| Colwell 2020 | Low | Low | Low | Low | Low | Low | Low | Low | Low | High | Low | High |  |
| Cramer 2015 | N/A | N/A | N/A | N/A | N/A | N/A | Low | Low | Low | High | Low | High |  |
| Cramer 2016 | N/A | N/A | N/A | N/A | N/A | N/A | Low | Low | SC | High | Low | High |  |
| Culos-Reed 2006 | N/A | N/A | N/A | N/A | N/A | N/A | SC | Low | Low | High | Low | High |  |
| Danhauer 2009 | N/A | N/A | N/A | N/A | N/A | N/A | SC | Low | Low | High | SC | High |  |
| Dhruva 2015 | N/A | N/A | N/A | N/A | N/A | N/A | Low | Low | Low | High | Low | High |  |
| Duzgun 2020 | N/A | N/A | N/A | N/A | N/A | N/A | SC | Low | SC | High | SC | High |  |
| Eaton 2021 | N/A | N/A | N/A | N/A | N/A | N/A | Low | Low | Low | High | Low | High |  |
| Eaton 2022 | low | low | low | SC | low | SC | N/A | N/A | N/A | N/A | N/A | N/A |  |
| Elkins 2008 | N/A | N/A | N/A | N/A | N/A | N/A | Low | Low | Low | High | Low | High |  |
| Elyasi 2021 | SC | Low | SC | SC | SC | SC | SC | Low | SC | High | SC | High |  |
| Eyigor 2018 | N/A | N/A | N/A | N/A | N/A | N/A | SC | Low | High | High | SC | High |  |
| Fink 2023 | low | low | low | SC | low | SC | N/A | N/A | N/A | N/A | N/A | N/A |  |
| Foley 2010 | N/A | N/A | N/A | N/A | N/A | N/A | Low | Low | Low | High | Low | High |  |
| Franco 2020 | N/A | N/A | N/A | N/A | N/A | N/A | Low | Low | High | High | SC | High |  |
| Gao R 2018 | N/A | N/A | N/A | N/A | N/A | N/A | Low | Low | SC | High | Low | High |  |
| Gregoire 2020 | N/A | N/A | N/A | N/A | N/A | N/A | Low | Low | Low | High | Low | High |  |
| Gross 1995 | N/A | N/A | N/A | N/A | N/A | N/A | Low | Low | High | High | Low | High |  |
| Gu 2024 | Low | Low | Low | SC | Low | SC | N/A | N/A | N/A | N/A | N/A | N/A |  |
| Hanser 2006 | N/A | N/A | N/A | N/A | N/A | N/A | Low | Low | Low | High | SC | High |  |
| Harper 2001 | Low | Low | Low | SC | Low | SC | N/A | N/A | N/A | N/A | N/A | N/A |  |
| Hidderley 2004 | N/A | N/A | N/A | N/A | N/A | N/A | SC | Low | High | High | SC | High |  |
| Ho 2016 (bms) | Low | Low | Low | SC | Low | SC | N/A | N/A | N/A | N/A | N/A | N/A |  |
| Ho 2016 (dance) | N/A | N/A | N/A | N/A | N/A | N/A | Low | Low | Low | High | Low | High |  |
| Hoogland 2018 | N/A | N/A | N/A | N/A | N/A | N/A | SC | Low | SC | High | Low | High |  |
| Huang YY 2007 | N/A | N/A | N/A | N/A | N/A | N/A | SC | Low | Low | High | SC | High |  |
| Ioannou 2022 | SC | low | low | SC | low | SC | N/A | N/A | N/A | N/A | N/A | N/A |  |
| Irwin 2017 | Low | Low | Low | Low | Low | Low | N/A | N/A | N/A | N/A | N/A | N/A |  |
| Jain 2012 | Low | Low | Low | Low | Low | Low | N/A | N/A | N/A | N/A | N/A | N/A |  |
| Janusek 2019 | Low | Low | Low | SC | Low | SC | N/A | N/A | N/A | N/A | N/A | N/A |  |
| Jaya 2020 | SC | Low | High | SC | SC | High | N/A | N/A | N/A | N/A | N/A | N/A |  |
| Jensens-Johansen 2013 | Low | Low | Low | SC | Low | SC | N/A | N/A | N/A | N/A | N/A | N/A |  |
| Johns 2016 | Low | Low | Low | SC | Low | SC | N/A | N/A | N/A | N/A | N/A | N/A |  |
| Jong 2018 | N/A | N/A | N/A | N/A | N/A | N/A | SC | High | Low | High | Low | High |  |
| Juarez 2024 | N/A | N/A | N/A | N/A | N/A | N/A | High | Low | Low | High | Low | High |  |
| Kahveci 2025 | Low | Low | Low | SC | Low | SC | N/A | N/A | N/A | N/A | N/A | N/A |  |
| Kiecolt-Glaser 2014 | N/A | N/A | N/A | N/A | N/A | N/A | Low | Low | Low | High | Low | High |  |
| Kim 2005 | N/A | N/A | N/A | N/A | N/A | N/A | Low | Low | Low | High | SC | High |  |
| Kim 2015 | N/A | N/A | N/A | N/A | N/A | N/A | Low | Low | Low | High | SC | High |  |
| Kingston 2015 | N/A | N/A | N/A | N/A | N/A | N/A | SC | Low | Low | High | SC | High |  |
| Knoerl 2022 | N/A | N/A | N/A | N/A | N/A | N/A | SC | Low | Low | High | Low | High |  |
| Koca 2022 | N/A | N/A | N/A | N/A | N/A | N/A | high | low | low | high | SC | high |  |
| Kremerer 2023 | SC | Low | SC | Low | Low | SC | High | Low | Low | High | Low | High |  |
| Krischer 2007 | N/A | N/A | N/A | N/A | N/A | N/A | Low | Low | SC | High | Low | High |  |
| Larkey 2015 | Low | Low | Low | Low | Low | Low | N/A | N/A | N/A | N/A | N/A | N/A |  |
| Larkey 2022 | Low | Low | Low | Low | Low | Low | N/A | N/A | N/A | N/A | N/A | N/A |  |
| Lau BHP 2020 | Low | Low | Low | Low | Low | Low | N/A | N/A | N/A | N/A | N/A | N/A |  |
| Lee 2017 | N/A | N/A | N/A | N/A | N/A | N/A | Low | Low | Low | High | SC | High |  |
| Lee 2020 | N/A | N/A | N/A | N/A | N/A | N/A | SC | Low | Low | High | SC | High |  |
| Leite 2021 | Low | Low | SC | SC | Low | SC | N/A | N/A | N/A | N/A | N/A | N/A |  |
| Lengacher 2009 | N/A | N/A | N/A | N/A | N/A | N/A | Low | Low | Low | High | Low | High |  |
| Lengacher 2016 | N/A | N/A | N/A | N/A | N/A | N/A | High | Low | Low | High | Low | High |  |
| Lengacher 2025 | Low | Low | Low | SC | Low | SC | High | Low | Low | High | Low | High |  |
| Li WJ 2020 | N/A | N/A | N/A | N/A | N/A | N/A | Low | High | Low | High | Low | High |  |
| Li XM 2012 | N/A | N/A | N/A | N/A | N/A | N/A | Low | Low | Low | High | SC | High |  |
| Lima 2020 | Low | Low | Low | SC | SC | High | N/A | N/A | N/A | N/A | N/A | N/A |  |
| Lin MF 2011a | Low | Low | Low | SC | SC | SC | Low | Low | Low | High | SC | High |  |
| Lin MF 2011b | N/A | N/A | N/A | N/A | N/A | N/A | Low | Low | Low | High | Low | High |  |
| Liossi 2001 | N/A | N/A | N/A | N/A | N/A | N/A | Low | Low | High | High | SC | High |  |
| Liu CJ 2008 | N/A | N/A | N/A | N/A | N/A | N/A | Low | Low | High | High | SC | High |  |
| Liu J 2016 | N/A | N/A | N/A | N/A | N/A | N/A | Low | Low | High | High | SC | High |  |
| Liu Q 2022 | low | low | high | SC | low | high | low | low | high | high | low | high |  |
| Liu W 2022 | N/A | N/A | N/A | N/A | N/A | N/A | SC | Low | Low | High | Low | High |  |
| Liu X 2020 | N/A | N/A | N/A | N/A | N/A | N/A | SC | Low | High | High | SC | High |  |
| Liu Z 2022 | N/A | N/A | N/A | N/A | N/A | N/A | Low | Low | SC | High | Low | High |  |
| Lopez 2023 | N/A | N/A | N/A | N/A | N/A | N/A | high | low | SC | high | low | high |  |
| Lu N 2023 | N/A | N/A | N/A | N/A | N/A | N/A | SC | low | low | high | low | high |  |
| Ma 2024 | N/A | N/A | N/A | N/A | N/A | N/A | Low | Low | Low | High | Low | High |  |
| Malik 2024 | Low | Low | SC | Low | SC | SC | N/A | N/A | N/A | N/A | N/A | N/A |  |
| Mantoudi 2020 | High | Low | Low | SC | SC | High | N/A | N/A | N/A | N/A | N/A | N/A |  |
| Masoume 2021 | Low | Low | Low | SC | Low | SC | Low | Low | Low | High | Low | High |  |
| McCombie 2023 | low | low | SC | SC | low | SC | N/A | N/A | N/A | N/A | N/A | N/A |  |
| Mi Ra 2017 | Low | Low | Low | SC | SC | SC | N/A | N/A | N/A | N/A | N/A | N/A |  |
| Milbury 2013 | N/A | N/A | N/A | N/A | N/A | N/A | Low | Low | Low | High | Low | High |  |
| Milbury 2019 | Low | Low | Low | High | High | High | N/A | N/A | N/A | N/A | N/A | N/A |  |
| Molassiotis 2002 | Low | Low | Low | SC | Low | SC | N/A | N/A | N/A | N/A | N/A | N/A |  |
| Molassiotis 2021 | N/A | N/A | N/A | N/A | N/A | N/A | Low | Low | SC | High | SC | High |  |
| Monti 2013 | Low | Low | SC | SC | Low | SC | N/A | N/A | N/A | N/A | N/A | N/A |  |
| Mosher 2024 | N/A | N/A | N/A | N/A | N/A | N/A | Low | Low | SC | High | Low | High |  |
| Nakamura 2013 | Low | Low | Low | SC | Low | SC | N/A | N/A | N/A | N/A | N/A | N/A |  |
| Napoles 2020 | N/A | N/A | N/A | N/A | N/A | N/A | SC | Low | Low | High | Low | High |  |
| Ngamkham 2017 | N/A | N/A | N/A | N/A | N/A | N/A | Low | Low | SC | High | Low | High |  |
| Nguyen 2023 | N/A | N/A | N/A | N/A | N/A | N/A | Low | Low | SC | High | Low | High |  |
| Nissen 2019 | N/A | N/A | N/A | N/A | N/A | N/A | Low | Low | Low | High | Low | High |  |
| Noh Gie 2011 | N/A | N/A | N/A | N/A | N/A | N/A | SC | Low | High | High | SC | High |  |
| Nuzhath 2024 | N/A | N/A | N/A | N/A | N/A | N/A | SC | Low | SC | High | Low | High |  |
| O'Callaghan 2012 | N/A | N/A | N/A | N/A | N/A | N/A | Low | Low | Low | High | Low | High |  |
| Pan YN 2017 | N/A | N/A | N/A | N/A | N/A | N/A | SC | Low | SC | High | Low | High |  |
| Park 2020 | N/A | N/A | N/A | N/A | N/A | N/A | Low | Low | Low | High | Low | High |  |
| Potthoff 2012 | Low | Low | Low | SC | Low | SC | N/A | N/A | N/A | N/A | N/A | N/A |  |
| Pouy 2018 | N/A | N/A | N/A | N/A | N/A | N/A | Low | Low | Low | High | Low | High |  |
| Puig 2006 | N/A | N/A | N/A | N/A | N/A | N/A | Low | Low | Low | High | SC | High |  |
| Qiao Li 2018 | N/A | N/A | N/A | N/A | N/A | N/A | Low | Low | High | High | SC | High |  |
| Qin J 2016 | N/A | N/A | N/A | N/A | N/A | N/A | SC | Low | Low | High | Low | High |  |
| Rabinowitch 2023 | SC | SC | SC | Low | SC |  | N/A | N/A | N/A | N/A | N/A | N/A |  |
| Raghavendra 2009 | Low | Low | Low | SC | Low | SC | N/A | N/A | N/A | N/A | N/A | N/A |  |
| Rao 2009 | SC | High | SC | SC | Low | High | N/A | N/A | N/A | N/A | N/A | N/A |  |
| Robins 2013 | Low | Low | High | SC | Low | High | Low | Low | High | High | Low | High |  |
| Sabo 1996 | N/A | N/A | N/A | N/A | N/A | N/A | High | Low | High | High | Low | High |  |
| Sarenmalm 2017 | Low | Low | Low | Low | Low | Low | N/A | N/A | N/A | N/A | N/A | N/A |  |
| Schroder 2022 | Low | Low | SC | SC | Low | SC | N/A | N/A | N/A | N/A | N/A | N/A |  |
| Shan MS 2019 | N/A | N/A | N/A | N/A | N/A | N/A | Low | Low | SC | High | SC | High |  |
| Shao D 2020 | N/A | N/A | N/A | N/A | N/A | N/A | Low | Low | SC | High | Low | High |  |
| Sharpe 2019 | SC | Low | SC | Low | SC | SC | N/A | N/A | N/A | N/A | N/A | N/A |  |
| Shelley 2014 | N/A | N/A | N/A | N/A | N/A | N/A | SC | Low | Low | High | Low | High |  |
| Shergill 2022 | N/A | N/A | N/A | N/A | N/A | N/A | Low | Low | Low | High | low | High |  |
| Smith 2001 | N/A | N/A | N/A | N/A | N/A | N/A | Low | Low | Low | High | Low | High |  |
| Sohl 2022 | N/A | N/A | N/A | N/A | N/A | N/A | Low | Low | SC | High | Low | High |  |
| Song QH 2013 | N/A | N/A | N/A | N/A | N/A | N/A | SC | Low | Low | High | SC | High |  |
| Speca 2000 | N/A | N/A | N/A | N/A | N/A | N/A | Low | Low | Low | High | Low | High |  |
| Sun J 2018 | N/A | N/A | N/A | N/A | N/A | N/A | SC | Low | SC | High | SC | High |  |
| Targ 2002 | Low | Low | Low | SC | Low | SC | N/A | N/A | N/A | N/A | N/A | N/A |  |
| Taylor 2018 | N/A | N/A | N/A | N/A | N/A | N/A | Low | Low | SC | High | SC | High |  |
| Thyme 2009 | N/A | N/A | N/A | N/A | N/A | N/A | Low | Low | SC | High | Low | High |  |
| Vargas-Roman 2022 | N/A | N/A | N/A | N/A | N/A | N/A | SC | Low | Low | High | Low | High |  |
| Vaziri 2017 | N/A | N/A | N/A | N/A | N/A | N/A | SC | High | Low | High | SC | High |  |
| Victorson 2020 | N/A | N/A | N/A | N/A | N/A | N/A | Low | Low | High | High | Low | High |  |
| Victorson 2024 | Low | Low | SC | Low | Low | SC | N/A | N/A | N/A | N/A | N/A | N/A |  |
| Walker 1997 | N/A | N/A | N/A | N/A | N/A | N/A | SC | Low | Low | High | SC | High |  |
| Wan YH 2009 | N/A | N/A | N/A | N/A | N/A | N/A | Low | Low | High | High | SC | High |  |
| Wen 2022 | N/A | N/A | N/A | N/A | N/A | N/A | low | low | low | high | SC | High |  |
| Wen MH 2016 | N/A | N/A | N/A | N/A | N/A | N/A | SC | Low | SC | High | Low | High |  |
| Wong 2024 | N/A | N/A | N/A | N/A | N/A | N/A | low | Low | Low | High | Low | High |  |
| Wu GX 2020 | N/A | N/A | N/A | N/A | N/A | N/A | Low | Low | High | High | SC | High |  |
| Wu Xiuying 2015 | N/A | N/A | N/A | N/A | N/A | N/A | Low | Low | Low | High | SC | High |  |
| Wurtzen 2013 | N/A | N/A | N/A | N/A | N/A | N/A | Low | Low | SC | High | Low | High |  |
| Xu HY 2018 | SC | Low | Low | SC | Low | SC | SC | Low | Low | High | Low | High |  |
| Xu YL 2018 | N/A | N/A | N/A | N/A | N/A | N/A | SC | Low | SC | High | SC | High |  |
| Yang 2010 | Low | Low | Low | SC | SC | SC | Low | Low | Low | High | SC | High |  |
| Yao 2022 | N/A | N/A | N/A | N/A | N/A | N/A | low | low | low | high | low | high |  |
| Yildrim 2024 | N/A | N/A | N/A | N/A | N/A | N/A | SC | Low | Low | High | SC | High |  |
| Ying W 2019 | N/A | N/A | N/A | N/A | N/A | N/A | Low | Low | Low | High | Low | High |  |
| You M 2020 | N/A | N/A | N/A | N/A | N/A | N/A | Low | Low | SC | High | Low | High |  |
| Yun X 2014 | N/A | N/A | N/A | N/A | N/A | N/A | Low | Low | High | High | SC | High |  |
| Zetzl 2021 | N/A | N/A | N/A | N/A | N/A | N/A | Low | Low | Low | High | Low | High |  |
| Zhang 2023 | N/A | N/A | N/A | N/A | N/A | N/A | SC | Low | Low | High | Low | High |  |
| Zhang JY 2017 | N/A | N/A | N/A | N/A | N/A | N/A | Low | Low | Low | High | Low | High |  |
| Zhang R 2020 | N/A | N/A | N/A | N/A | N/A | N/A | SC | Low | SC | High | SC | High |  |
| Zhang XA 2011 | N/A | N/A | N/A | N/A | N/A | N/A | High | Low | High | High | SC | High |  |
| Zhao PT 2008 | N/A | N/A | N/A | N/A | N/A | N/A | Low | Low | SC | High | SC | High |  |
| Zhi 2021 | N/A | N/A | N/A | N/A | N/A | N/A | SC | Low | SC | High | Low | High |  |
| Zhou KN 2011 | N/A | N/A | N/A | N/A | N/A | N/A | Low | Low | Low | High | SC | High |  |
| Zhu WH 2012 | N/A | N/A | N/A | N/A | N/A | N/A | Low | Low | SC | High | SC | High |  |
| Zhuang XQ 2020 | N/A | N/A | N/A | N/A | N/A | N/A | Low | Low | Low | High | Low | High |  |
| Zhuo W 2017 | N/A | N/A | N/A | N/A | N/A | N/A | SC | Low | High | High | SC | High |  |

Table 1 presents the risk of bias of the 182 studies included in the systematic review according to the type of control intervention. N/A = not applicable; SC = some concerns.
